# Positive and Negative Effects under Light Illumination in Halide Perovskites

**DOI:** 10.1002/smsc.202400028

**Published:** 2024-05-12

**Authors:** Guijun Zhang, Qianwen Wei, Mehri Ghasemi, Guangsheng Liu, Juan Wang, Binjian Zhou, Jingjing Luo, Yu Yang, Baohua Jia, Xiaoming Wen

**Affiliations:** ^1^ International Joint Research Center for Optoelectronic and Energy Materials School of Materials and Energy Yunnan University Kunming Yunnan 650091 China; ^2^ Centre for Atomaterials and Nanomanufacturing RMIT University Melbourne VIC 3000 Australia

**Keywords:** light soaking effects, metal halide perovskites, negative effects, physical mechanisms, positive effects

## Abstract

Metal halide perovskites (MHPs) have excellent characteristics and present great potential in a broad range of applications such as solar cells, light‐emitting diodes, and photodetectors. However, the light stability of devices remains an unresolved issue and has received great research attention. Under light illumination, MHPs exhibit various anomalous phenomena, such as photoluminescence (PL) enhancement, defect curing, PL blinking, and phase segregation. These phenomena are commonly considered intimately correlated with the performance and stability of MHP devices. In recent years, significant efforts have been made experimentally and theoretically toward understanding the physical origins of these anomalous effects. However, most research focuses on negative effects while the positive effects are mostly ignored. Herein, the positive effects and the negative effects of light soaking in MHPs are systematically discussed with a classification of the correlated physical mechanisms by specifically focusing on variation occurring in timescale from second to hour, corresponding to the unique ionic–electronic interaction. This intends to provide a new insight into ion effects on excellent properties of perovskites, and deep physical understanding of charge‐carrier and ion dynamics in perovskite, and theoretical guidelines for the fabrication of high‐quality and stability perovskite‐based photovoltaic and photoelectric devices.

## Graphical abstract

For the first time, light soaking effects are separated into positive and negative effects simultaneously occurring in halide perovskites. The positive effects are correlated with the lattice/sublattice effect, while the negative effects are ascribed to the charge and defect effects of mobile ions. The apparent effect is determined by the intensity of illumination and nature of the material.

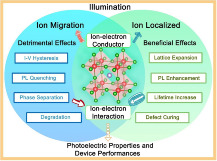

## 1 Introduction

In just over a decade of research, the power conversion efficiency (PCE) of metal halide perovskites (MHPs) devices has experienced unprecedented growth. In 2009, Kojima first used this material to prepare dye‐sensitized solar cells, with an efficiency of 3.8%.^[^ ^1^ ^]^ Currently, it has enjoyed skyrocketing developments of up to 26%.^[^ ^2^ ^]^ However, the inefficient operational stability of the systems is still a challenge yet to overcome, which has been recognized as the bottleneck for the broad commercial application of perovskite solar cells (PSCs).^[^ ^3^, ^4^ ^]^ Many external factors can cause the degradation of perovskite materials, such as moisture, oxygen, illumination, electric field, and heat.^[^ ^5^, ^6^, ^7^ ^]^ Several crucial questions related to the underlying mechanisms of the operational function of the devices including unstable output characteristics (light soaking phenomena) as well as the role of the ions in the soft lattice, are poorly understood.^[^ ^8^ ^]^

Light soaking effect (LSE), which generally describes a temporary device performance change after a certain duration of light illumination, has been commonly observed in various kinds of solar cells.^[^ ^9^, ^10^ ^]^ The severity of the LSE is reported to vary from laboratory to laboratory and remains the subject of intense debate regarding the underlying mechanism. In contrast, the instability of the test samples under light and the sample evolution proposed by Rao et al. may also lead to the unreplicability of the experimental data, which is also why the LSE observed in each laboratory is inconsistent.^[^ ^11^ ^]^ There are inconsistencies between most of the previously published articles in this area of research. The underlying physics of LSE is complicated, and the contributed effects can be reflected as either positive or negative, which can intimately correlate with the performance and stability of the perovskite devices. Therefore, a thorough comprehension of this ambiguous phenomenon and the underlying causes are of utmost importance to improve the performance and stability of PSC. Previously, LSE has been presented mainly as a negative characteristic with its negative contribution in PSCs *I*–*V* hysteresis,^[^ ^12^ ^]^ phase segregation,^[^ ^13^ ^]^ photoluminescence (PL) blinking,^[^ ^14^ ^]^ degradation, and ultimately unsatisfactorily poor stability. These observations were attributed to the contribution of the mobile ions which dominantly act as the nonradiative recombination centers in the system. By contrast, most of the positive effects (such as defect tolerance,^[^ ^15^ ^]^ defect curing/healing,^[^ ^16^ ^]^ which results in illumination‐enhanced PL and longer carrier lifetime^[^ ^17^ ^]^) come from nondefective ions (lattice, sublattice) in which the components of electric charge, and the defective character, commonly observed in mobile ions, are absent. It is necessary to note that these positive effects are responsible for superior optoelectronic properties and the outstanding performance of perovskite devices. Yang et al. systematically reviewed the recent advances in developing effective approaches and strategies to mitigate or eliminate the negative effects of LSE in PSCs.^[^ ^8^ ^]^ However, the positive effects of LSE on materials and devices have been considerably ignored.

Based on thermodynamics and statistical physics, LSE occurs randomly with statistical probability relative to their threshold that depends on the decomposition, fabrication, ambience, and stimulus conditions. Generally, the positive and negative effects occur simultaneously under given illumination conditions. Localized hot ion as part of lattice or sublattice results in positive effects, which mostly occurs at low illumination. In contrast, at high illumination intensity, the mobile ions are activated and generated, mostly responsible for the negative effect. Moreover, the process is reversible. Therefore, in the same material people usually observe the positive effect at low illumination and the negative effect at high illumination.^[^ ^18^ ^]^ Therefore, studying the phenomenon and mechanism of LSE in perovskites and fully understanding their positive and negative effects have important theoretical guidance and practical value for various applications of perovskite devices.

In this review, we summarize the LSE in MHPs by systematically describing the underlying physical mechanisms. We first classify the LSE into positive effects and negative effects to provide a comprehensive understanding of LSE in MHPs. The positive effects include light effects on the microstructure and chemical composition of MHPs, enhanced PL intensity, and prolonged carrier lifetime. The positive effects of light soaking on PSCs, photoelectric detectors, and light‐emitting devices (LED) are also reviewed. Then, the negative effects mostly involving optical properties such as PL quenching, film quality including phase separation, and degradation of perovskite materials, the performance of corresponding devices, and *I*–*V* hysteresis phenomenon in solar cells were discussed. Finally, we emphasize the importance and particularity of ion effects on excellent properties of perovskites and their devices. This review proposes a new insight into ion effects on excellent properties of perovskites, and a deep physical understanding of charge‐carrier and ion dynamics in perovskite.

## 2 The Contribution of Composition and Defects in LSEs

The composition of perovskite materials can seriously affect the LSE phenomenon. Paul Fassl et al. presented a detailed study on how fractional, and quite possibly unintentional, changes in the stoichiometry of the precursor solution have an enormous effect on the properties (surface composition and energetics, PL, crystallinity, energetic disorder, etc.) of perovskite films with seemingly similar, or the same, microstructure and as a result on the device performance and stability.^[^ ^19^ ^]^ MHPs are a class of typical ionic semiconductors.^[^ ^20^, ^21^ ^]^ A three‐dimensional (3D) MHP is represented by the chemical formula of ABX_3_, where A is an organic cation such as methylammonium (MA^+^), formamidinium (FA^+^), or an inorganic cation such as Cs^+^, B is a divalent metal cation such as Pb^2+^ or Sn^2+^, and X is a halide ion of I^−^ or Br^−^.^[^ ^22^, ^23^ ^]^ The adjustable crystal lattice of MHPs allows multi‐ion alloying or partial doping at the same site.^[^ ^24^ ^]^ For example, B‐site can be either alloyed or partially doped with other ions such as Sb^3+^,^[^ ^25^ ^]^ Zn^2+^,^[^ ^26^ ^]^ Cd^2+^,^[^ ^27^ ^]^ In^2+^,^[^ ^28^ ^]^ Mn^2+^,^[^ ^29^ ^]^ Ca^2+^,^[^ ^30^ ^]^ Ce^2+^,^[^ ^31^ ^]^ and Bi^3+^.^[^ ^32^, ^33^ ^]^ By alloying a bigger size ion at the A‐site such as a long organic cation, the structural dimension of MHPs can be transformed from 3D to low‐dimensional structures of 2D, 1D, or 0D.^[^ ^34^ ^]^ Among them, the most common 2D halide perovskites are divided into Ruddlesden–Popper (RP) phase, Dion–Jacobson (DJ) phase, and the alternating cation phase.^[^ ^24^, ^27^, ^35^, ^36^ ^]^ In addition, through alloying, the stability of the crystal structure and the optical properties can be intentionally modulated.^[^ ^37^, ^38^ ^]^ By varying the ratio of the X‐site halides or B‐site divalent cations the optical bandgap of MHPs can be effectively tuned covering the light spectrum from visible to near‐infrared (NIR) region highly demanding for a wide range of optoelectronic applications.^[^ ^39^ ^]^

**Figure 1:**
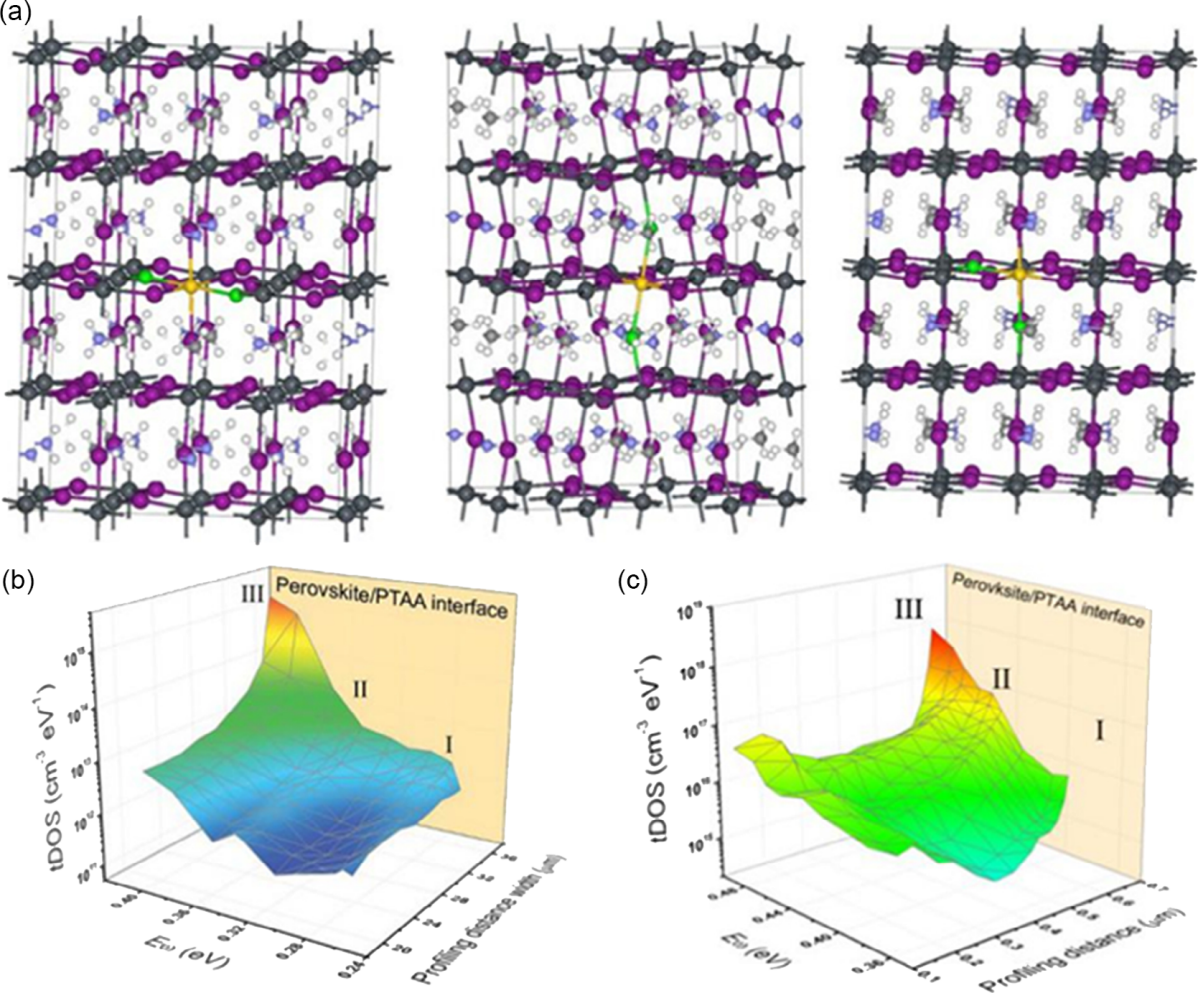
a) The three types of position of PbI_2_ vacancy in MAPbI_3_. Adapted with permission.^[^ ^44^ ^]^ Copyright 2014, American Chemical Society. b) Spatial and energy mapping of the densities of trap states in the single crystal and c) MAPbI_3_ film. Adapted with permission.^[^ ^47^ ^]^ Copyright 2020, The American Association for the Advancement of Science.

Usually, MHP thin films can be fabricated by the low‐cost solution process, in which the formation of many grain boundaries and defects are inevitable.^[^ ^40^ ^]^ Although vacuum deposition as an elaborated method can also be used, defects are still unavoidable.^[^ ^41^ ^]^ Even in the case of the MHP single crystals which are supposed to be free of defects, the generation of defects is abundant.^[^ ^42^ ^]^ The characteristics of these defects and ions species affect the initial optical properties of perovskite. Numerous studies have shown that there are many intrinsic point defects (a type of Schottky defect) located inside the bulk of MHPs such as neutral PbI_2_ or MAI vacancies in the MAPbI_3_ perovskite.^[^ ^43^ ^]^ Take the common PbI_2_ vacancies as an example, the formation energy of PbI_2_ vacancies is very low in the MHP structure.^[^ ^44^ ^]^ As shown in **Figure** **1**a, the formation energies are 27, 73, and 44 meV for three types positions, respectively. In the soft lattice of MHPs, the configuration of point defects is not energetically stable. Under illumination, ion migration can be promoted due to lower formation energy, which leads to the transition from energetically stable configurations to metastable configurations.^[^ ^45^, ^46^ ^]^ It is well known that defect states usually can act as the recombination center or act like the doped ions, negative for efficient carrier transporting and the PCE of PSCs. These defect states are in fact the trap states that deleteriously induce electron trapping. It was suggested that shallow traps normally form from point defects and distribute throughout the entire MAPbI_3_ structure, whereas deep traps mostly form from surface defects like dangling bonds located at the surface both of the MAPbI_3_ film and single crystal (Figure 1b,c).^[^ ^47^ ^]^ Moreover, ion migration in perovskite is dominated by surface defects and leads to the generation of new defects.^[^ ^48^ ^]^

In recent years, many unique anomalous slow phenomena have been observed in perovskite films such as hysteresis in current–voltage characteristics,^[^ ^49^ ^]^ or PL quenching under bias electric field,^[^ ^50^ ^]^ which occur in timescale of seconds to hours. These phenomena are usually considered to be correlated to the ionic contribution of MHPs, because ions intrinsically have extremely slow response time as well as much lower mobility compared with electron carriers response, which is within nanoseconds.^[^ ^49^, ^51^ ^]^ Similarly, some phenomena belong to LSEs such as PL intensity enhancement, prolonged carrier lifetime, or PL quenching were observed,^[^ ^52^, ^53^ ^]^ which are strongly associated with ions behavior and defects characteristics in MHPs. The behavior of ions in perovskite depends on the light illumination condition. Under light illumination, electrons were generated from high‐energy photons in the UV region, with energy much higher than the optical bandgap of the MHPs, which results in the formation of hot carriers. Afterward, these hot carriers experience ultrafast cooling and relax to the conduction band (CB) via emission of longitudinal optical phonons. Through the emission of acoustic phonon into lattice, the ions in lattice can also obtain energy through ion‐phonon scattering.^[^ ^54^ ^]^ These interactions between the ions and the lattice of the MHPs after obtaining energy under light illumination should be the key parameters governing the LSEs in these materials. These ion dynamics and behind mechanisms will be discussed in next section.

## 3 Positive Effects of LSE

The modification of chemical composition and microstructure can enhance the photoelectric properties of perovskite materials, such as defect mitigation and increased PL quantum yield. Recognizing the significance of these factors, this section reviews the positive impacts of LSE on the microstructure and chemical composition of perovskites. These effects encompass enlarged grain size, perovskite alloying, defect reduction, enhanced grain boundary properties, and so on.

### 3.1 MHPs Microstructure and Chemical Composition

**Figure 2:**
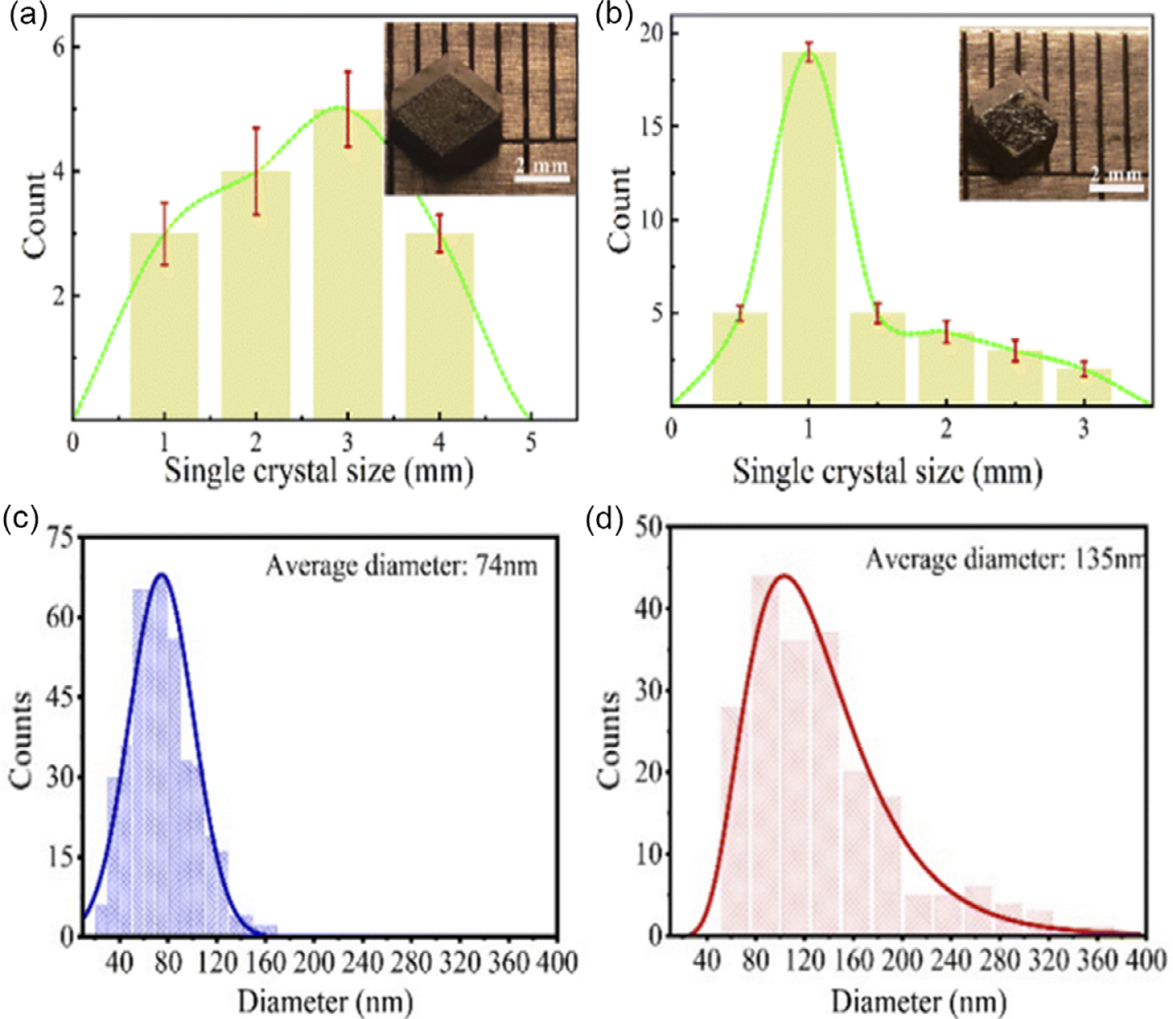
a) The statistical data of the size and number of MAPbI_3_ single crystals in their precursor solutions under light and b) dark conditions, respectively, the light intensity of which is 64 mW cm^−2^. The insert images are the MAPbI_3_ single crystal grown from its precursor solution after 36 h. Adapted with permission.^[^ ^58^ ^]^ Copyright 2022, The Royal Society of Chemistry. c) MAPbI_3_ films obtained by spin‐coating under different illumination intensities 0 mW cm^−2^, d) 52 mW cm^−2^), and the statistics of the grain diameter distribution of the MAPbI_3_ microcrystalline in films. Adapted with permission.^[^ ^58^ ^]^ Copyright 2022, The Royal Society of Chemistry.

**Figure 3:**
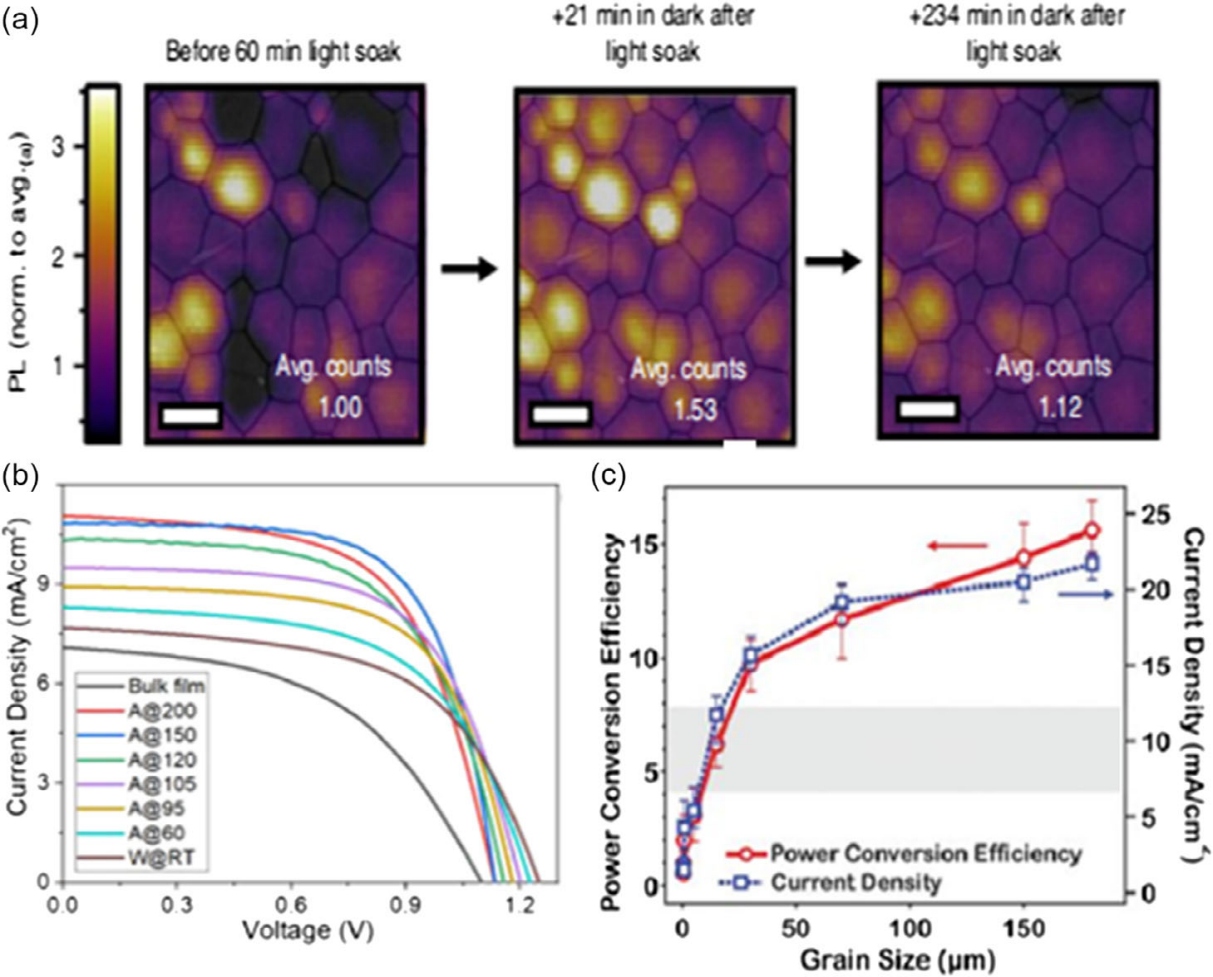
a) Before light soaking, and after exposing the entire film to simulated sunlight (AM 1.5, 100 mW cm^−2^) for 60 min and leaving in the dark for 21 and 234 min (all images have the same PL intensity scale normalized to the average PL intensity in (a), scale bars, 1 mm).^[^ ^59^ ^]^ Copyright 2016, Springer Nature. b) The *J*–*V* curves of the different grain size champion photovoltaic cells under 100 mW cm^−2^ of solar light AM 1.5G, 60 represents 60° annealing. Adapted with permission.^[^ ^60^ ^]^ Copyright 2021, American Chemical Society. c) Average overall PCE (left) and *J*_SC_ (right) as a function of crystalline grain size. Adapted with permission.^[^ ^61^ ^]^ Copyright 2015, The American Association for the Advancement of Science.

The most effect of illumination on the lattice of MHPs is lattice expansion. It has been confirmed in experiments that exposure to light can cause the hybrid perovskite lattice to expand by 1.4%,^[^ ^55^ ^]^ which in turn increases the efficiency of solar cells by several percentage points. Tsai et al. attributed the LSE in FA_0.7_MA_0.25_Cs_0.05_PbI_3_ solar cells to a lattice expansion caused by weakened covalent bonds between Pb–I under light illumination, which releases the lattice strain and lowers the energetic barriers at perovskite and contact interfaces. However, this conclusion was challenged by Rolston et al.^[^ ^56^ ^]^ who claimed that the lattice expansion is mainly caused by the radiative heating. Further, grain size can also affect the luminescent properties of perovskite materials. Tian et al. observed that light‐induced PL enhancement in the MAPbI_3_ strongly depends on the expansion of the crystal size.^[^ ^57^ ^]^ Thereby, whether the lattice expansion is purely a thermal effect is still controversial; but large lattice expansion under continuous light exposure is an accepted fact. Wang et al. indicated that the external illumination on the growth of perovskite single crystals, where the considerably larger size MAPbI_3_ single crystals can be obtained under illumination conditions (**Figure** **2**a,b).^[^ ^58^ ^]^ Likewise, the preparation of the MAPbI_3_ films under illumination by a one‐step spin coating yields to the formation of thin films with much increased crystal size and improved quality. (Figure 2c,d). Accordingly, the PCE of the corresponding solar cell increased from 15.2% for MHP thin film synthesized with no light illumination to 17.7% for the illumination‐treated MAPbI_3_ thin film. DeQuilettes et al. also observed that an obvious increase in grain size of MHP thin film after illumination, with an ultimate enhancement of the PL intensity (**Figure** **3**a).^[^ ^59^ ^]^

It was suggested that the grain size is closely related to the performance of PSCs. Hu et al. tuned the grain size of CsPbBr_1.5_I_1.5_ films derived from nanocrystals by annealing at different temperatures to investigate the corresponding charge transport properties.^[^ ^60^ ^]^ The correlation between phase segregation, grain size, mobility, trap density, carrier lifetime and the corresponding solar cell performance is unraveled. Figure 3b shows the current density‐voltage (*J*–*V*) curves of solar cells made of perovskite films with different grain sizes by controlling the annealing temperature that clearly shows the enhancement of the solar cell performance over grain‐size enlargement. Similarly, Nie et al fabricated the mixed‐halide organometallic perovskite photovoltaic devices using the hot‐casting technique in a planar device architecture.^[^ ^61^ ^]^ They explored the effects of different grain sizes on the performance and the current density of the fabricated solar cell device (Figure 3c) that showed the positive effect of the grain size enlargement on the performance of the PSCs, in consistent with previous reports. Yan et al. utilized a laser‐annealing method to obtain high‐crystallinity perovskite films, benefiting from the laser‐induced high‐temperature gradient and in consequence leading to the selective growth of large perovskite grains.^[^ ^62^ ^]^

**Figure 4:**
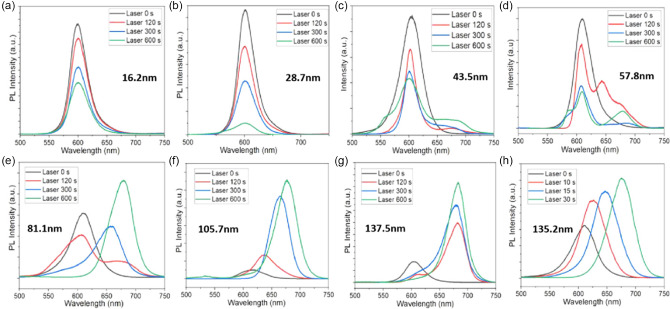
Normalized PL emission spectra of different grain size a) 16.2 nm, b) 28.7 nm, c) 43.5 nm, d) 57.8 nm, e) 81.1 nm, f) 105.7 nm, g) 157.5, and h) 135.2 nm under light irradiation (10 mW cm^−2^). Adapted with permission.^[^ ^60^ ^]^ Copyright 2021, American Chemical Society.

More importantly, the grain size has a significant impact on the optical response of the MHP thin film and the corresponding device to LSE. Performing time‐dependent PL on different films, Hu et al. showed different responses of the PL spectra for CsPbBr_1.5_I_1.5_ perovskite films with different crystal sizes under continuous illumination^[^ ^60^ ^]^ (**Figure** **4**). The PL intensity of perovskite films with grain size less than 60 nm (Figure 4a–d) decreased gradually with the increase of irradiation time, but this phenomenon was reversed for MHP thin films with the grain size higher than 60 nm (Figure 4e–h). Experiments have shown that lattice expansion may affect charge carrier recombination.^[^ ^55^ ^]^ The effect of lattice expansion on carrier recombination has been studied, showing a slight decrease in the radiation recombination rate of the photogenerated charge carriers.^[^ ^63^, ^64^ ^]^

**Figure 5:**
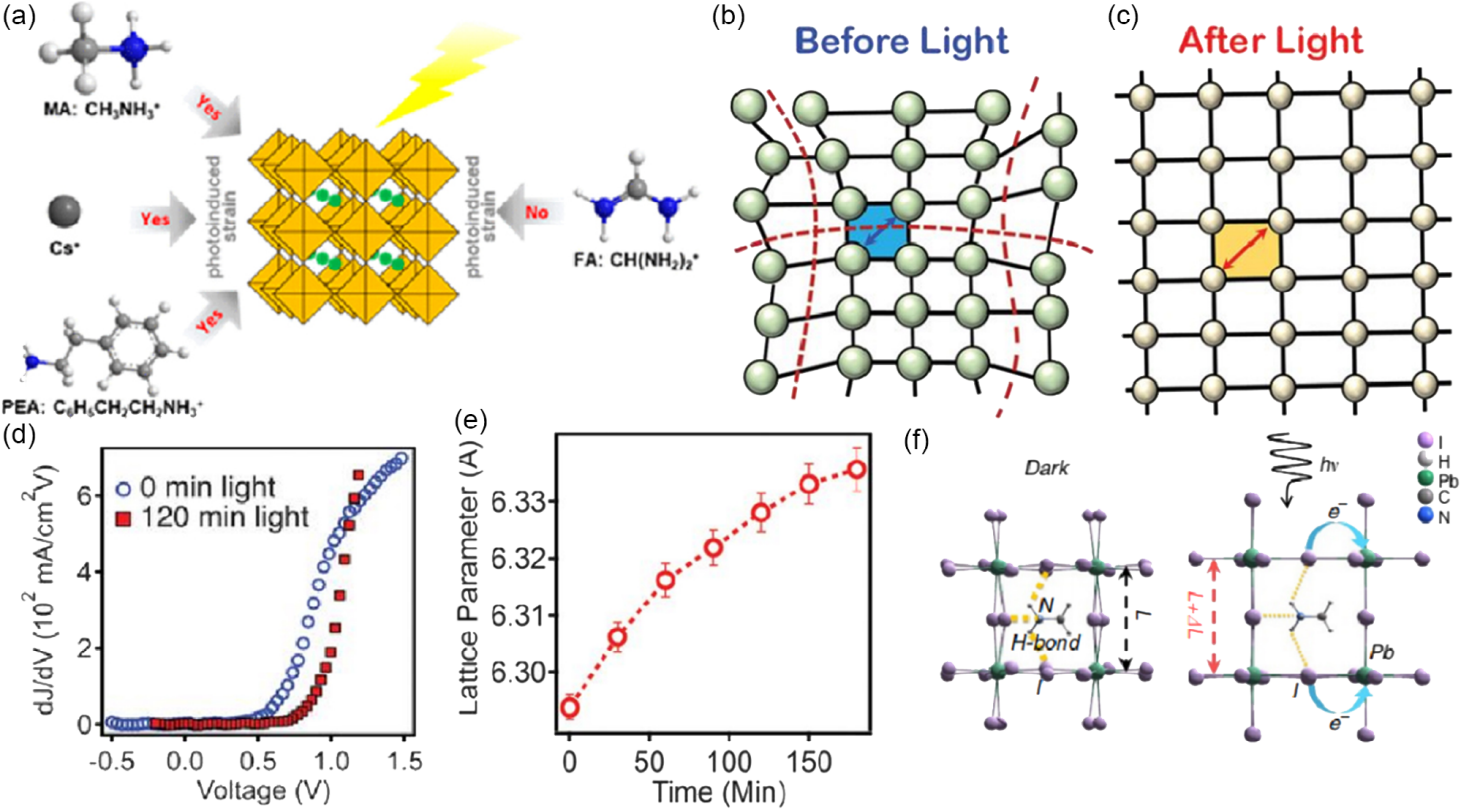
a) Diagram of different situations in which the lattice size changes when light and heat act alone. Adapted with permission.^[^ ^65^ ^]^ Copyright 2021, American Chemical Society. b,c) Schematic describing the crystal structure change before illumination (local distortion) and after illumination (lattice expansion). Adapted with permission.^[^ ^55^ ^]^ Copyright 2018, The American Association for the Advancement of Science. d) First‐order derivative of the *J*–*V* curves before and after 2 h of light soaking. Adapted with permission.^[^ ^55^ ^]^ Copyright 2018, The American Association for the Advancement of Science. e) Lattice constant as a function of illumination time. Error bars indicate the error from peak fitting. Adapted with permission.^[^ ^55^ ^]^ Copyright 2018, The American Association for the Advancement of Science. f) Mechanism for the giant photostriction in MAPbI_3_. Schematic illustrations (not to scale) show that the weakening of the hydrogen bonding between the amine group and the iodine ion by photo‐generated carriers leads to the lattice expansion. Adapted with permission.^[^ ^67^ ^]^ Copyright 2016, Springer Nature.

Liu et al. revealed that the changes in lattice size occur differently when light and heat act alone. Among four cations of MA, FA, Cs, and PEA studied in their work, FA is different from the other three cations, where FA‐based MHP (FAPbI_3_) does not show lattice expansion upon illumination while the other three cations exhibit considerable lattice expansion. In contrast, all four cations (FA, MA, Cs, and PEA)‐based MHPs exhibit a lattice expansion upon heating (**Figure** **5**a).^[^ ^65^ ^]^ Performing rigorous first‐principles calculations for a prototypical hybrid‐perovskite FAPbI_3_, Zhang et al. showed that 1% lattice expansion can reduce the nonradiative capture coefficient by one order of magnitude.^[^ ^66^ ^]^ They found the suppressed nonradiative trapping is not caused by the changes of the bandgap or defect transition level due to lattice expansion, but originates from enhanced defect relaxation associated with charge‐state transitions in the expanded lattice.

Tsai et al. suggested that the LSE behavior is related to the light‐induced lattice expansion rather than the ion migration, the longer the illumination time, the larger the grain size (Figure 5b,c).^[^ ^55^ ^]^ They also observed the increase of PCE from 18.5% to 20.5% under continuous light illumination of 1 sun (100 mW cm^−2^) in a device with mixed‐cation pure‐halide perovskite (Figure 5d). They furthermore investigated the performance of solar cells under continuous illumination and found that *V*_oc_, FF as well as the lattice constant (Figure 5e) increase with increasing illumination time. They rationalized that on microscopic scale, illuminating the hybrid perovskite films with photon energies greater than the bandgap develops electron–hole pairs in the material. The photogenerated electrons in the CB can populate bonding states, whereas holes in the valence band (VB) vacate antibonding states. Both processes can weaken covalent bonds and lead to either less‐distorted Pb—I—Pb bonds or elongation of the Pb—I bond, which causes lattice expansion. Besides, Yang et al. indirectly attributed the postillumination changes in the lattice to the weakening of the hydrogen bonding between MA and iodide (Figure 5f).^[^ ^67^ ^]^ In contrast, Rolston et al. showed that by controlling the temperature of the perovskite film under both dark and illumination conditions, the mechanism for lattice expansion is in fact fully consistent with heat‐induced thermal expansion during illumination.^[^ ^56^ ^]^ which means that there is no consensus on the physical mechanism of lattice expansion of perovskite under continuous illumination.

**Figure 6:**
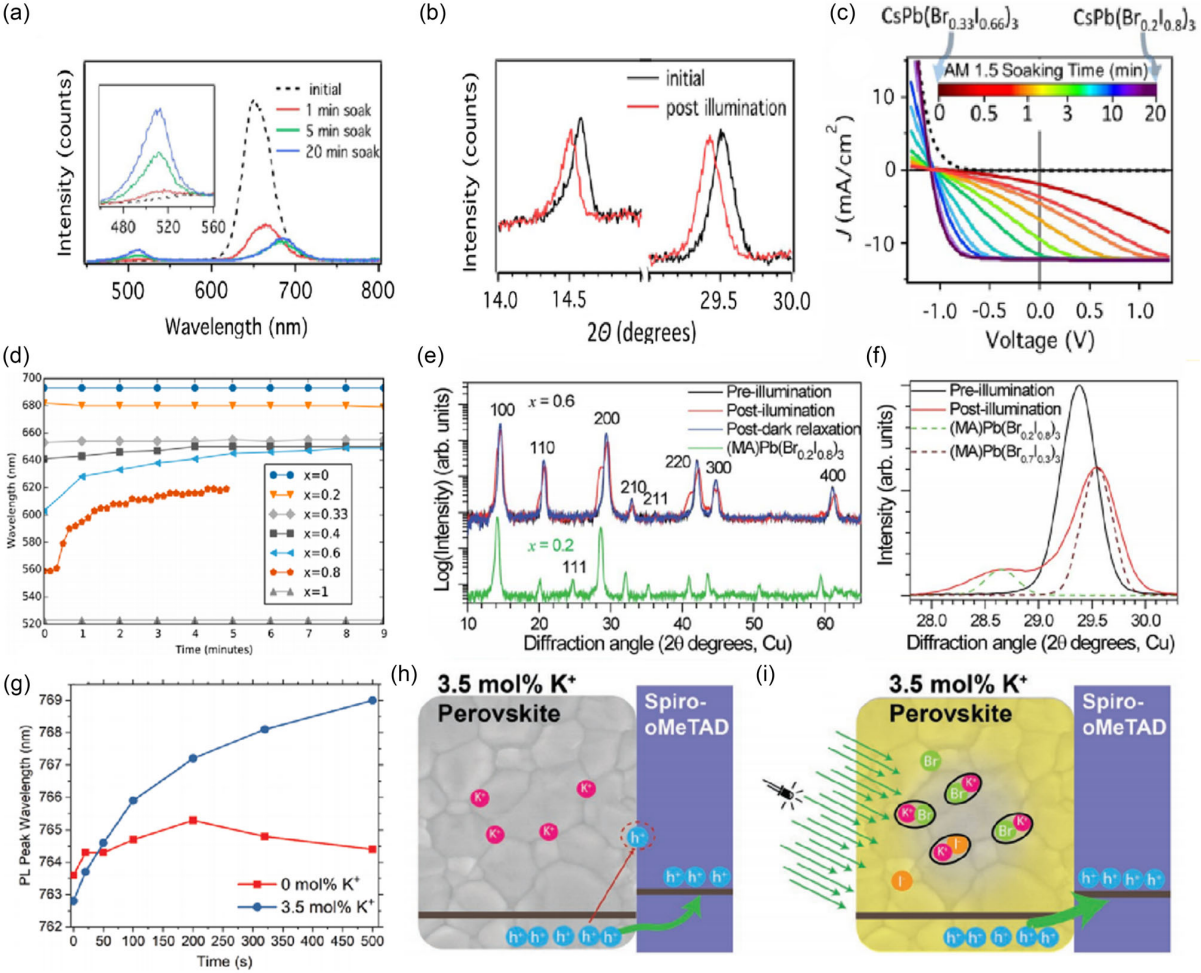
a) PL spectra of a CsPbBrI_2_ film on glass for different times of light exposure. Adapted with permission.^[^ ^68^ ^]^ Copyright 2017, American Chemical Society. b) XRD spectra of ITO/TiO_2_/CsPbBrI_2_/Spiro device sealed with Kapton film. Adapted with permission.^[^ ^68^ ^]^ Copyright 2017, American Chemical Society. c) Relationship between perovskite dealloying and the efficiency of solar cell devices. Adapted with permission.^[^ ^68^ ^]^ Copyright 2017, American Chemical Society. d) PL peak position as a function of time for CsPb(BrxI_1−*x*_)_3_ materials under ≈1 sun illumination. Adapted with permission.^[^ ^69^ ^]^ Copyright 2016, American Chemical Society. e) XRD pattern of an *x* = 0.6 film before (black) and after (red) and *x* = 0.2 film (green) white‐light soaking for 5 min at ≈50 mW cm^−2^, and after 2 h in the dark (blue). f) The 200 XRD peak of an *x* = 0.6 film before (black) and after (red) white‐light soaking for 5 min at 50 mW cm^−2^. XRD patterns of an *x* = 0.2 film (dashed green) and an *x* = 0.7 film (dashed brown). Adapted with permission.^[^ ^70^ ^]^ Copyright 2015, The Royal Society of Chemistry. g) PL peak positions of 0 and 3.5 mol% K^+^ perovskite films plotted to excitation time. Adapted with permission.^[^ ^71^ ^]^ Copyright 2019, John Wiley and Sons. Schematic of the passivation effect of K^+^‐doped perovskite triggered by illumination:3.5 mol% K^+^ perovskite in h) the dark and i) during light soaking. Adapted with permission.^[^ ^71^ ^]^ Copyright 2019, John Wiley and Sons.

There are also some other causes, such as the passivation effect of the formation of KBr‐like compounds, oxidized Pb—O bond networks, and light‐induced halide dealloying. Niezgoda et al. investigated the performance evolution of CsPbBrI_2_ solar cells under continuous illumination (AM 1.5).^[^ ^68^ ^]^ The devices exhibited dramatic performance enhancement, resulting from the light‐induced dealloying of CsPbBrI_2_ that improved the collection of holes. Continuous illumination caused the dealloying of CsPbBrI_2_ film into I‐ and Br‐rich regions which is evident both in the ≈35 nm redshift of the initial PL peak (emission from increasingly I‐rich phase) and the emergence of higher‐energy peak near 520 nm (emission from formation of Br‐rich regions, **Figure** **6**a). Figure 6b shows further evidence of dealloying in CsPbBrI_2_ through a shift in X‐ray diffraction (XRD) peak position as a result of light soaking. The shift to lower 2*θ* after light soaking is indicative of the formation of large I‐rich regions in PV devices during illumination. They found that the light‐induced dealloying of CsPbBrI_2_ was positive in achieving high‐efficiency solar cells (Figure 6c). This is of particular relevance to CsPb(Br_*x*_I_1−*x*_)_3_ compound that exhibits light‐induced dealloying (Figure 6d).^[^ ^69^ ^]^ Hoke et al. reported the reversible, light‐induced transformations in MAPb(Br_*x*_I_1−*x*_)_3_.^[^ ^70^ ^]^ Light illumination causes a splitting of XRD peaks, suggesting segregation from a single crystalline phase into two separate I‐rich and Br‐rich phases. Surprisingly, these photo‐induced changes are fully reversible (Figure 6e,f).

Wen et al. explored the mechanism of ion migration suppression in the perovskite film upon K^+^ doping.^[^ ^71^ ^]^ A gradual halide ion substitution of Br^−^ by I^−^ occurred upon continuous illumination in the K^+^‐doped perovskite film, significantly different from the case without K^+^ doping. Figure 6g depicts PL peak positions of a MHP film doped with 3.5 mol% K^+^ showing a significant redshift relative to the excitation time, while for the case of the pure MHP film the variation of the PL peak position over illumination is not significant. A notable reduction in the density of the interface trap states was also recorded by K^+^ doping, which led to a significant increase in the hole extraction efficiency, and therefore the improvement of PCE of the PSCs. They propose that the passivation effect by K^+^ doping must be activated by light illumination, after which Br^−^ anions are bonded to K^+^ to form immobile KBr‐like compounds (Figure 6h,i). These compounds not only eliminate the mobile halide ion defects in perovskite films but also suppress ion migration, resulting in improved stability and hysteresis‐free PSCs.

In addition, the presence of oxygen has also been shown to enhance PL,^[^ ^72^, ^73^ ^]^ but Haque et al. proposed the formation of a superoxide species that initiates the degradation of MAPbI_3_.^[^ ^74^ ^]^ Brenes et al. showed that as MAPbI_3_ is exposed to light, increased humidity can lead to higher PL quantum efficiencies.^[^ ^75^ ^]^ Godding et al. proposed a comprehensive mechanism for the reactivity of the archetypal perovskite, MAPbI_3_, in ambient conditions. They established the formation of Pb—O bonds by hydrogen peroxide as the key factor leading to perovskite photo‐brightening.^[^ ^76^ ^]^

**Figure 7:**
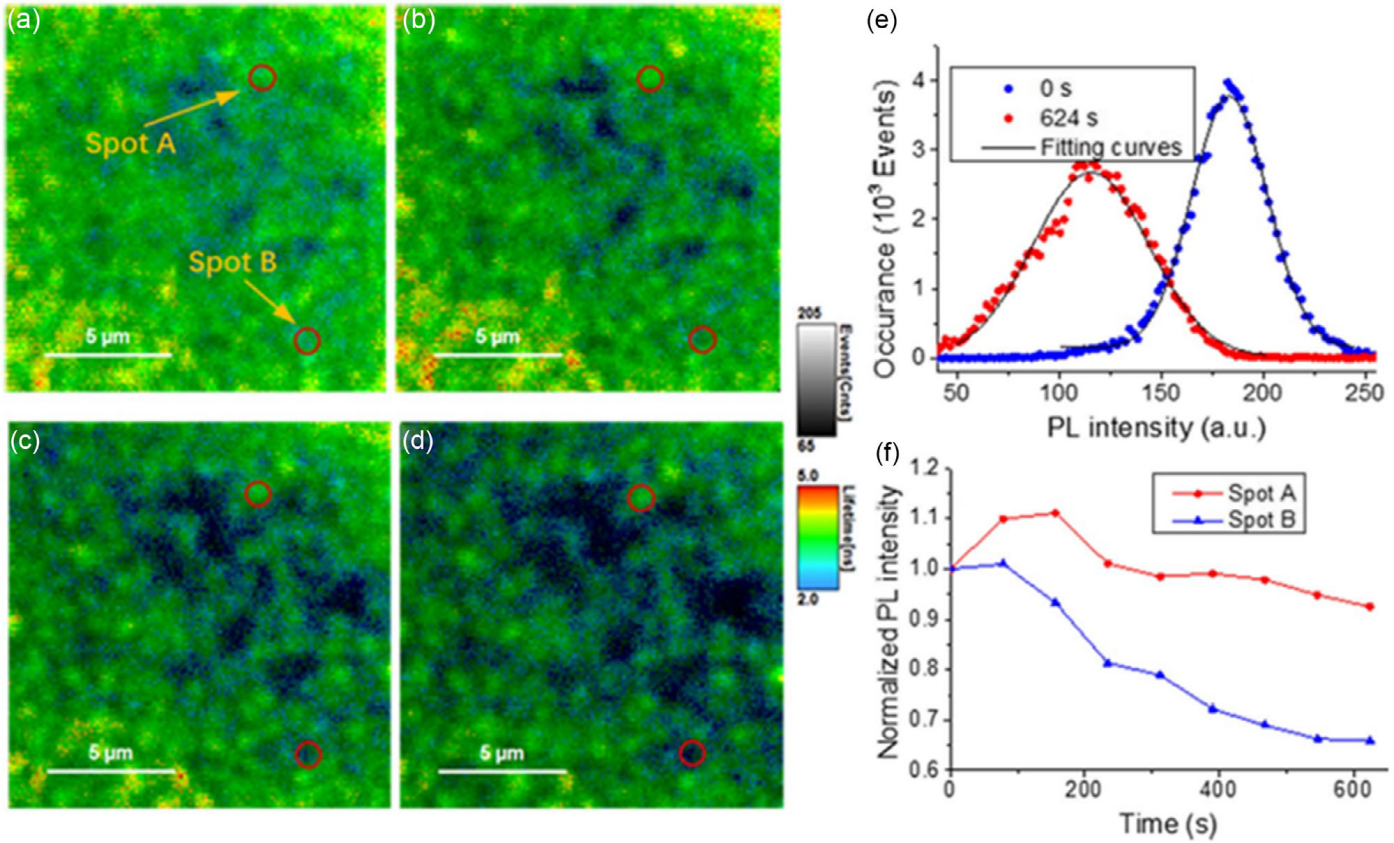
a) PL images of an MAPbI_3_ PSC at open‐circuit before, after b) 156 s, c) 390 s, and d) 624 s of light soaking. Spots A and B are marked by two red circles in the images. e) The distribution of PL intensity over measured area before (blue) and after (red) light soaking and f) normalized PL intensity of the spot A (red) and spot B (blue) as a function of light soaking time. Adapted with permission.^[^ ^77^ ^]^ Copyright 2018, Elsevier.

It was suggested there was an apparent correlation between LSE and defects of perovskites. Wen et al. obtained successive images that show the spatial distribution of both PL intensity and PL lifetime on a small region (15 × 15 μm) of MAPbI_3_ thin film.^[^ ^77^ ^]^ In these images, the local brightness indicates PL intensity and color indicates the carrier lifetime. The original sample measured before light soaking shows a relatively uniform PL distribution (**Figure** **7**a). As light soaking progresses, however, PL uniformity gradually decreases (Figure 7b–d). Several bright spots appeared on the image after light soaking. Figure 7e shows PL intensity distributions over the measured area before (blue curve, Stage 0) and after (red curve, Stage 2) light soaking. The two curves are fitted by Gaussian function. As can be seen, the full width at half maximum (FWHM) increased from 43.9 to 66.7 after light soaking for 10 mins, where the wider distribution of FWHM indicates less uniformity of the perovskite thin film. To understand this change, the PL evolution of two spots in the sample (spot A which is brighter, and spot B which is dimer to start) was tracked which evidently shows the increase of the contrast between the selected spots over illumination. Plotting the localized PL intensities of the two spots against light soaking time (Figure 7f) showed that the initial increase of the spot A PL intensity in contrast to spot B. This is because preaccumulated positive ions in spot B faster annihilate, and further investigation shows that these dimmer spots such as spot B assigned to clusters of small grains. while spot A assigned to large grains.

Later, Ginger et al. demonstrated that the microstructure of perovskite films affects the local carrier lifetime and emission intensity.^[^ ^78^ ^]^ They found that the lifetime is much shorter and emission is much weaker at grain boundaries than in grains. The large fluctuation of emission intensity from grain to grain implies the variation of the trap states. Their study indicates that morphology influences the fundamental properties of perovskite films, which in return affects the properties of the solar cells. Nie et al. demonstrated a solution‐based hot‐casting technique to grow continuous, pinhole‐free thin films of organometallic perovskites with millimeter‐scale crystalline grains.^[^ ^61^ ^]^ They fabricated planar solar cells with efficiencies approaching 18%, with little cell‐to‐cell variability. The devices show a hysteresis‐free photovoltaic response, which had been a fundamental bottleneck for the stable operation of perovskite devices. Characterization and modeling attribute the improved performance to reduced bulk defects and improved charge carrier mobility in large‐grain devices. Shao et al. investigated how the perovskite film microstructure, such as grain boundaries and grain size, affects the light soaking in mixed‐halide PSCs.^[^ ^79^ ^]^ They found that the compact films with smaller grain sizes show a negligible LSE, which indicates that the trap density is reduced when grain boundaries are fused.

### 3.2 Enhanced PL Intensity and Prolonged Carrier Lifetime

The positive effects of LSE originate from immobile ions (sublattice, lattice), rather than mobile ions. Light illumination‐induced PL enhancement and prolonged carrier lifetime generally occur in the timescale of second to hour that well matches the slow ionic response, but cannot classify as photoelectronic dynamics that occurs in timescale of nanosecond to microsecond. With continuous light illumination, some energy can accumulate in the lattice/sublattice through electron‐lattice coupling, interpreted as defect tolerance and defect curing/healing. Simultaneously, mobile ions always exist in perovskites and the density depends on the detailed composition and fabrication process. With continuous illumination, mobile ions can also obtain extra energy, which can enhance their migration and thus could result in ion substitution, phase segregation, and PL quenching, which probably leads to a complicated.

**Figure 8:**
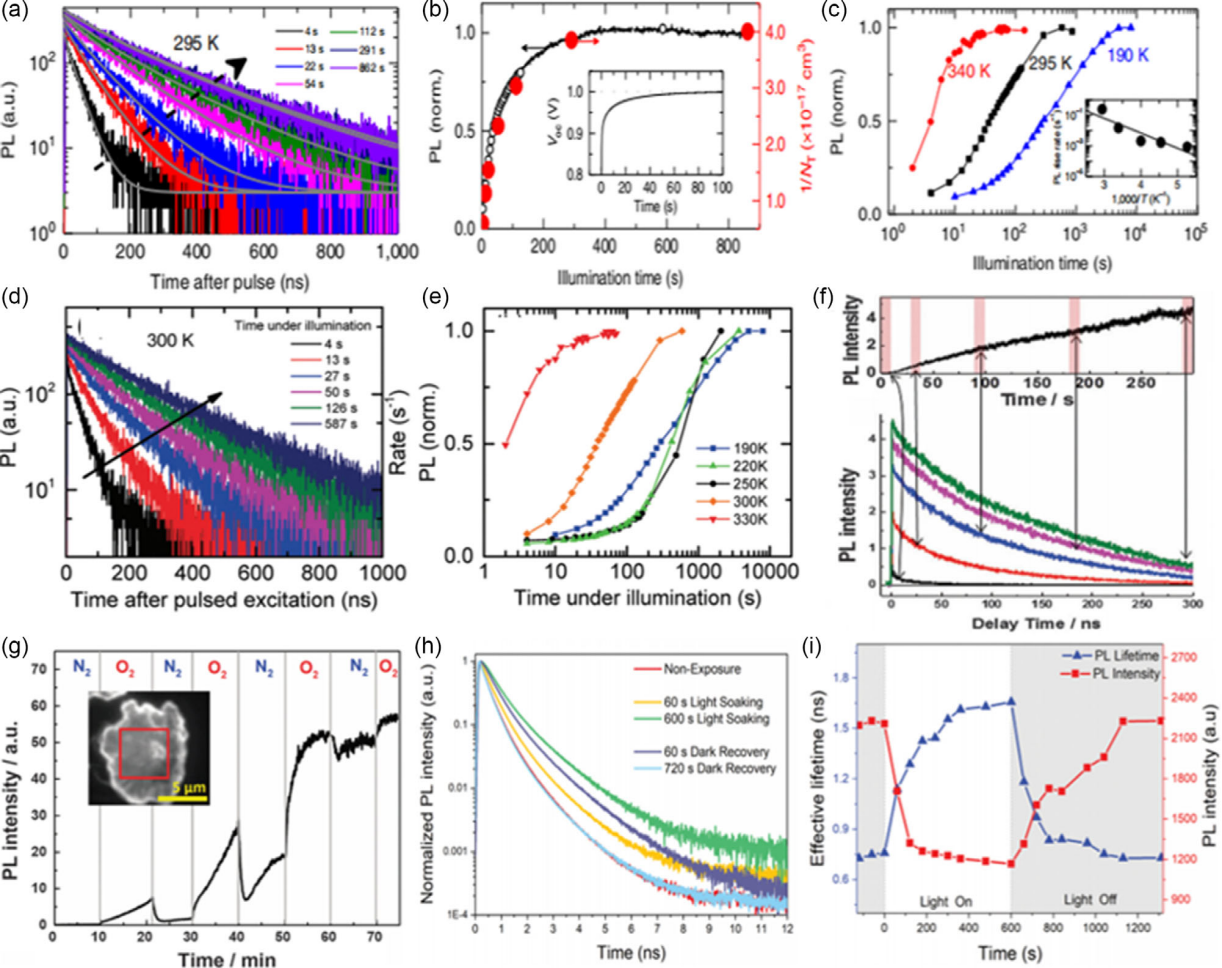
a) A series of time‐resolved PL decays from a MAPbI_3_ film measured over time under illumination. Adapted with permission.^[^ ^59^ ^]^ Copyright 2016, Springer Nature. b) PL intensity of MAPbI_3_ integrated over time under initial illumination. Adapted with permission.^[^ ^59^ ^]^ Copyright 2016, Springer Nature. c) The normalized integrated PL over time under illumination at different temperatures. Adapted with permission.^[^ ^59^ ^]^ Copyright 2016, Springer Nature. d) A series of time‐resolved PL decays from a MAPbI_3_ film measured in vacuum over time under illumination at 300 K. Adapted with permission.^[^ ^80^ ^]^ Copyright 2016, The Royal Society of Chemistry. e) The normalized integrated PL over time under illumination at different temperatures, acquired by integrating PL decays. Adapted with permission.^[^ ^80^ ^]^ Copyright 2016, The Royal Society of Chemistry. f) MAPbI_3_ film PL decay and PL steady‐state intensity as a function of light irradiation time. Adapted with permission.^[^ ^83^ ^]^ Copyright 2015, The Royal Society of Chemistry. g) The N_2_ and O_2_ atmosphere effect on the PL enhancement of MAPbI_3_. Adapted with permission.^[^ ^83^ ^]^ Copyright 2015, The Royal Society of Chemistry. h) PL decay traces in different illumination conditions from a CsPbI_3_ film. Adapted with permission.^[^ ^84^ ^]^ Copyright 2020, American Chemical Society. i) PL intensity and PL effective lifetime as a function of time for a CsPbI_3_ film. Adapted with permission.^[^ ^84^ ^]^ Copyright 2020, American Chemical Society.

It was found that under continuous light, the PL intensity^[^ ^17^, ^59^ ^]^ of MAPbI_3_ thin film and the carrier lifetime^[^ ^78^ ^]^ significantly improved. DeQuilettes et al. showed the time‐resolved PL of a neat MAPbI_3_ film (**Figure** **8**a), and PL intensity in Figure 8b, over a time period of 10 min under pulsed illumination in vacuum, which was correlated closely with the rise in open‐circuit voltage, the inset of Figure 8b.^[^ ^59^ ^]^ They also showed that the extent of the PL enhancements is influenced by different temperatures (Figure 8c). Besides, Mosconi et al. confirmed the lifetime and PL intensity of the MAPbI_3_ film continued to increase until reaching a stable state (Figure 8d), under continuous pulsed laser illumination.^[^ ^80^ ^]^ They discovered at lower temperatures (190 K), it takes a 2–3 order of magnitude longer time to reach stabilized emission than at higher temperatures (330 K) (Figure 8e). At present, there is no unified explanation for the mechanism of LSE in MAPbI_3_ thin films, but there is a basic consensus that defect states play an important role in it. Based on the defect state model proposed by Stranks et al.^[^ ^81^ ^]^ Mosconi et al.^[^ ^80^ ^]^ deduced that the defect state density in MAPbI_3_ thin films decreased from the initial 10^17^ to 10^16^ cm^−3^ before reaching a stable state, due to the role of defect states as nonradiative recombination centers in MAPbI_3_ thin films.^[^ ^81^ ^]^ Similarly, DeQuilettes et al. quantitatively analyzed the relationship between PL enhancement and the decrease of defect state density from initial 1.7 × 10^17^ cm^−3^ decreasing to 2.5 × 10^16^ cm^−3^ after 15 mins of light illumination.^[^ ^59^ ^]^ However, it has also been pointed out that when it comes to defects in MHPs, especially in thin films and single crystals, the method of calculating defect concentration may also be problematic.^[^ ^82^ ^]^

Tian et al. investigated a light‐induced PL enhancement in surface‐deposited MAPbI_3_ perovskites in detail using time‐resolved luminescence microscopy.^[^ ^83^ ^]^ PL intensity increases up to three orders of magnitude upon light illumination with an excitation power density of 0.01–1 W cm^−2^ (Figure 8f). Additionally, they found PL enhancement was more pronounced in oxygen gas compared to nitrogen (Figure 8g). Also, the PL intensity increases upon illumination for the PbCl_2_‐derived MAPbI_3−*x*_Cl_*x*_ perovskite, which was proposed to be a result of the stabilization of charge carrier trap states by photogenerated electrons and, possibly, light‐driven chemical changes in the semiconductor involving ionic motion.^[^ ^81^ ^]^ Defect curing of the perovskite film by light leads not only to PL intensity increase but also enhanced solar cell performance. Anaya et al. demonstrated that light illumination leads to reduced nonradiative carrier recombination and thus higher PL efficiency of MAPbX_3_ perovskites, which strongly depends on the level of molecular oxygen in the environment.^[^ ^73^ ^]^

To quantitively study light soaking and dark recovery processes, Wen et al. showed the time‐resolved PL decay traces and PL intensity, Figure 8h,i, as a function of time, corresponding to light soaking time of 0–600 s and dark recovery time of 600–1200 s. It is concluded: 1) the decrease of PL intensity during light soaking is accompanied by the increase of PL lifetime, while the dark recovery is the reverse process of light soaking; 2) the impact of light soaking is fully recoverable.^[^ ^84^ ^]^

**Figure 9:**
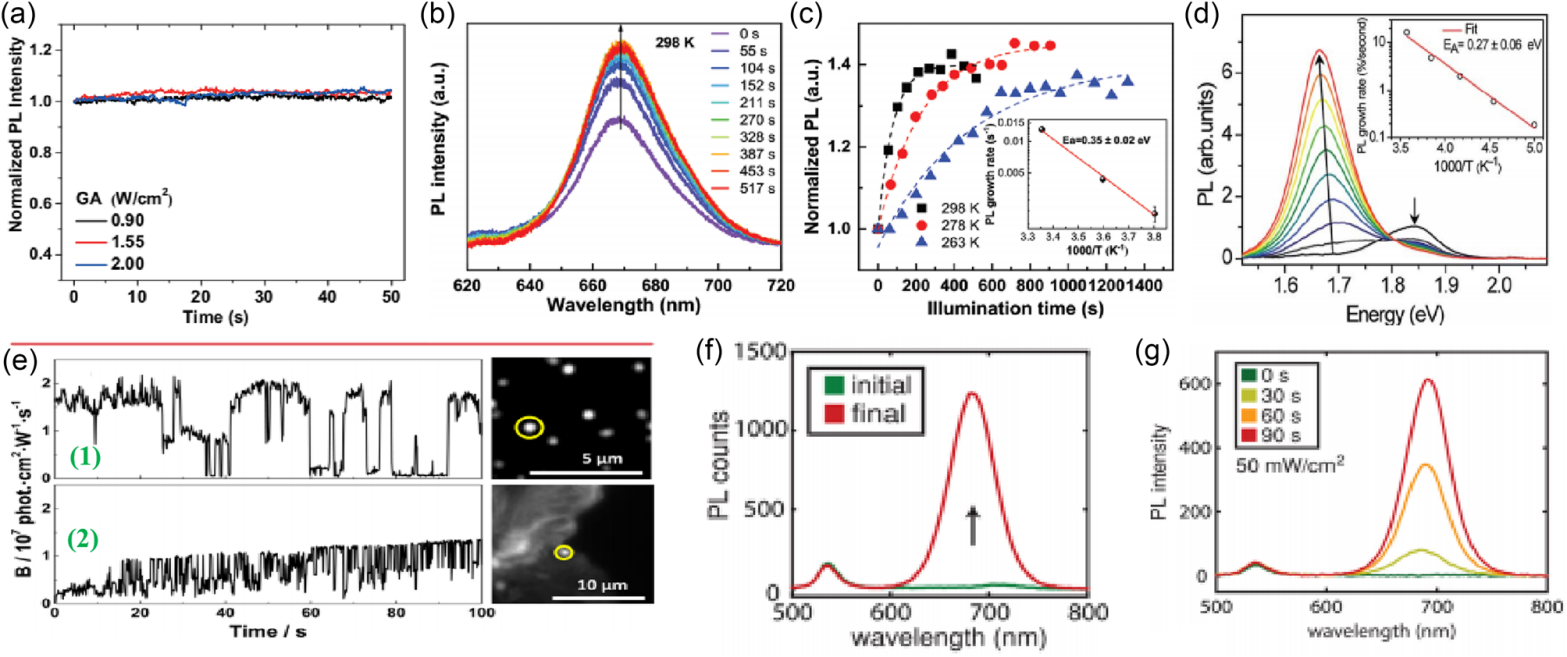
a) PL intensities over time of perovskite sample fabricated by gas‐assisted solution process technique under constant 470 nm laser excitations at (a) 0.90; 1.55 and 2.00 W cm^−2^. Adapted with permission.^[^ ^17^ ^]^ Copyright 2017, John Wiley and Sons. b) PL spectrums of the CsPb(I_0.8_Br_0.2_)_3_ perovskite film illuminated by 532 nm laser at 298 K. c) The normalized PL over illumination time at 298, 278, and 263 K. Adapted with permission.^[^ ^85^ ^]^ Copyright 2021, John Wiley and Sons. d) PL spectra of an (MA)Pb(Br_*x*_I_1−*x*_)_3_ with *x* = 0.4 thin film over 45 s in 5 s increments under 457 nm, 15 mW cm^−2^ light at 300 K. Adapted with permission.^[^ ^70^ ^]^ Copyright 2015, The Royal Society of Chemistry. e) PL transients in the units of brightness showing blinking of (1) a MAPbI_3_ nanocrystal and (2) a bright dot located on the top of a large MAPbI_3_ crystal. The locations of the objects are indicated in the images to the right. Adapted with permission.^[^ ^86^ ^]^ Copyright 2015, American Chemical Society. f) PL spectra of MAPb(I_0.1_Br_0.9_)_3_ before (green) and after (red) light soaking for 5 min. g) PL spectra after different light soaking times at 50 mW cm^−2^.^[^ ^87^ ^]^ Copyright 2017, American Chemical Society.

It has been found that LSE can significantly change the optical and electronic properties of perovskites and the performance of the corresponding device.^[^ ^17^, ^59^, ^85^ ^]^ Wen et al. investigated systematically the effect of light illumination on a well encapsulated MAPbI_3_ perovskites.^[^ ^17^ ^]^ Under a continuous constant illumination at low intensity, the PL exhibits either enhancement or constant (**Figure** **9**a); dependent on the detrapping rate and density of the defect states in the perovskite which in turn is determined by fabrication methods. Under continuous higher‐intensity illumination, PL quenching is observed with different thresholds relevant to the sample fabrication. This is ascribed to mobile ion accumulation resulting in increased electron/hole nonradiative recombination. Their research shows that different preparation techniques and excitation densities have a significant impact on the optical effect of MAPbI_3_ thin films under continuous illumination.

At room temperature, the intensity of PL peak at 668 nm gradually increased and finally stabilized after ≈300 s continuous laser exposure (Figure 9b,c), a signature of enhanced radiative recombination and reduced trap density. No peak splitting nor peak position shifting was observed, suggesting no composition changes during LS.^[^ ^85^ ^]^ Tian et al. observed thin films of the MAPbI_3_ possess highly spatially inhomogeneous and temporally fluctuating PL using fluorescence microscopy (Figure 9e).^[^ ^86^ ^]^ Besides the blinking, a clear increase in the average PL intensity can be seen in Figure 9d. This is because the PL quantum yield of the bulk crystal (which is the background of the blinking PL in this case) increases with light illumination.^[^ ^81^ ^]^ Note that MAPbI_3_ nanocrystals did not show any significant PL intensity increase upon light illumination. Local areas of large MAPbI_3_ conglomerates, as well as individual MAPbI_3_ nanocrystals, showed PL blinking behavior with exceptionally large amplitude. Hoke et al. found that the initial PL spectra for (MA)Pb(Br_*x*_I_1−*x*_)_3_ at low illumination intensities also continuously blue‐shift upon increasing bromide content.^[^ ^70^ ^]^ However, for perovskites with 0.2 < *x* < 1 they found that an additional PL peak forms at ≈1.68 eV and grows in intensity under continuous illumination. This PL spectral change is not dependent upon the spectrum or coherence of the light source because similar changes are observed with various white LEDs, 375 and 457 nm laser excitation, and red LED excitation (≈637 nm). Notably, these changes are reversible. Bischak et al. described how photoinduced phase separation in mixed halide hybrid perovskites can be mediated through strain‐induced phase separation at the locations of polarons‐photogenerated charge carriers and their accompanying lattice distortions.^[^ ^87^ ^]^ They observed significant PL enhancement after continuous illumination in Figure 9f,g.

### 3.3 The LSE Positive Effects on MHP‐Related Devices

The properties of the materials critically determine the performance of the associated devices. Perovskite materials have been widely studied for optoelectronic applications. Similarly, many researchers have observed the LSE when studying perovskite devices, such as PSCs, LEDs, and photodetectors.

#### 3.3.1 Solar Cells

For optoelectronic applications of MHPs, LSEs will inevitably occur in solar cell devices. Under working conditions, the long‐term output of perovskite‐based solar cells is reported to be unstable.^[^ ^88^ ^]^ LSE is one type of instability that occurs at the early stages of device operation and hinders the precise evaluation of device performance, which has been widely observed during the testing process of PSCs. Under LSE, PSCs experience a gradual increase of fill factor (FF), and open‐circuit voltage (*V*_oc_) with the extended light illumination time. Additionally, LSE also enhances the stability of solar cell devices.^[^ ^89^ ^]^ Deng et al. suggest that this improvement can be explained by directional drift of ions/vacancies in MPH materials driven by photovoltage‐induced electric field, forming p–*i*–n homojunction that improves the performance of MHP solar cells.^[^ ^90^ ^]^ In brief, LSE can be interpreted as the photoinduced structural transformation and properties variation in PSCs upon light illumination, which frequently results in an improved performance. Although widely observed in PSCs, the underlying mechanism behind the LSE is still under debate. Explanations such as trap state at the interface or bulk of perovskite, morphology, and the effect of ultraviolet light have all been proposed to account for this effect.^[^ ^78^, ^79^, ^81^, ^91^ ^]^

**Figure 10:**
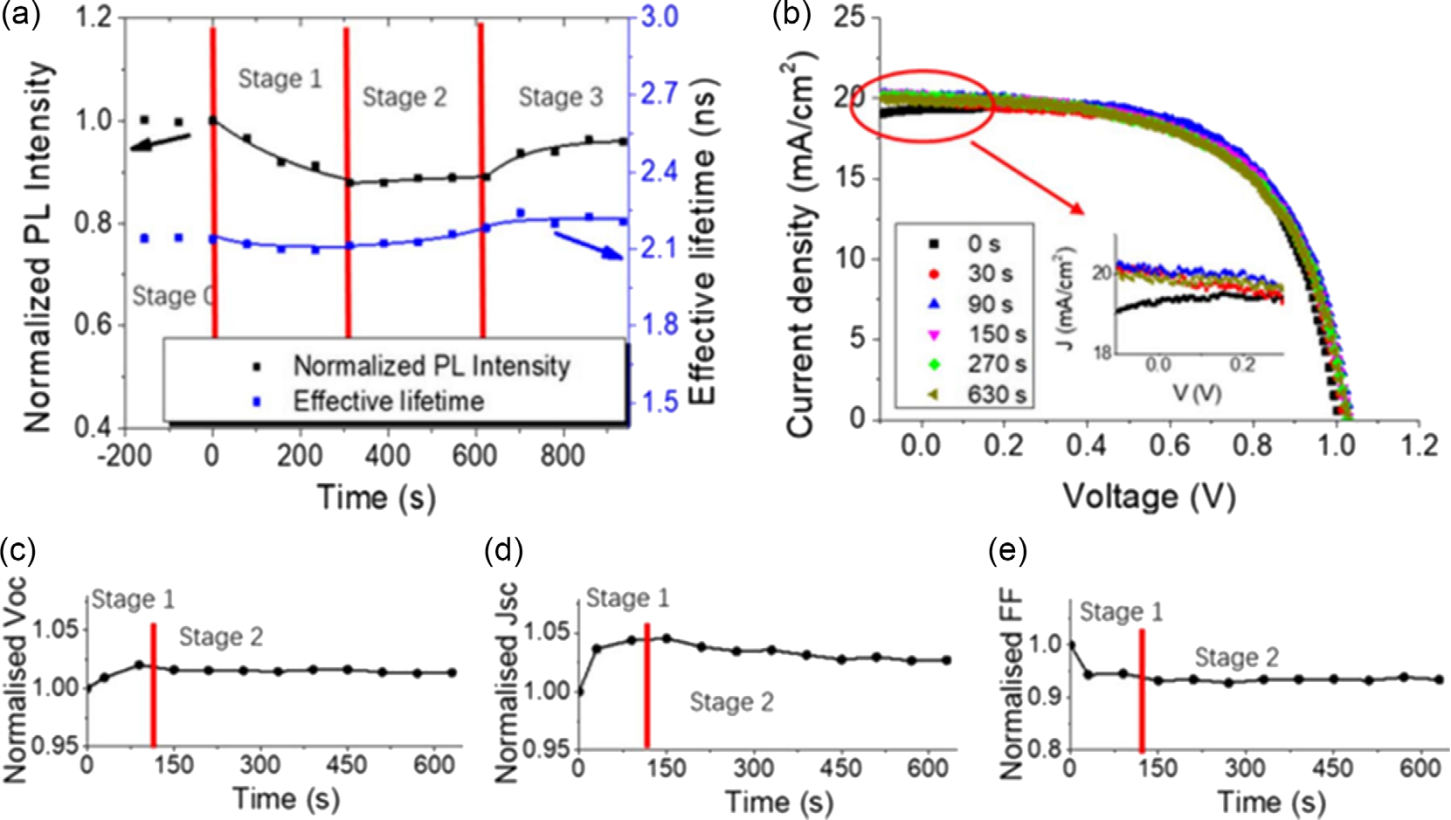
PL and *J*–*V* measurement results for a MAPbI_3_ PSC light soaked at short‐circuit condition. a) PL intensity and effective lifetime as functions of light soaking progress showing four different stages which are before light soaking (Stage 0), during light soaking (Stage 1 and 2), and after light soaking (Stage 3); b) *J*–*V* curves of the cell during light soaking process; c) *V*_oc_, d) *J*_sc_, and e) FF as functions of light soaking progress. Adapted with permission.^[^ ^77^ ^]^ Copyright 2018, Elsevier.

Wen et al. revealed that the bias voltage during light soaking results in different dynamic processes, which can be either positive or negative. They also suggested that the LSE predominantly occurs at the perovskite/transport layer interface due to ion accumulation.^[^ ^77^ ^]^ They investigated the LSE of PSCs at different voltages situations (open‐circuit and short‐circuit). The effect of light soaking at short‐circuit conditions on PL and *J*–*V* characteristics of MAPbI_3_ solar cells are shown in **Figure** **10**a,b. The significant difference in dynamic responses between short‐circuit and open‐circuit conditions indicates that the applied voltage plays an important role. Figure 10c–e shows the evolution of *V*_oc_, *J*_SC_, and FF during light soaking.

**Figure 11:**
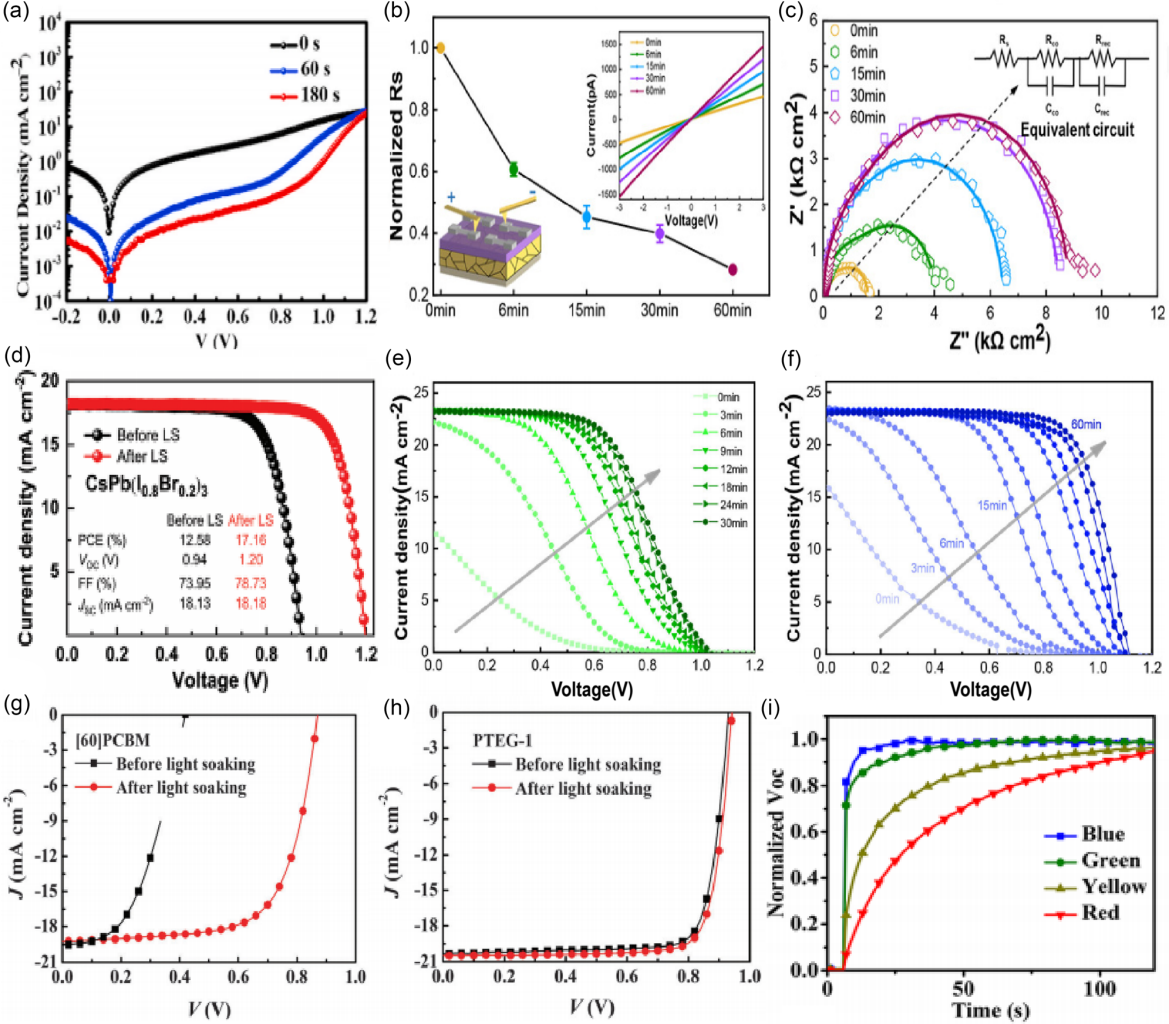
a) *J*–*V* curves after 0, 15, 30, and 45 min under light illumination. Adapted with permission.^[^ ^92^ ^]^ Copyright 2020, Elsevier. b) The normalized sheet resistance (*R*_sh_) of the devices (glass/perovskite/Spiro‐OMeTAD/Ag) measured by *I*–*V* characteristics. The inset spectra show the *I*–*V* curves of the device under different LS times. Adapted with permission.^[^ ^93^ ^]^ Copyright 2022, John Wiley and Sons. c) EIS under different LS times. Adapted with permission.^[^ ^93^ ^]^ Copyright 2022, John Wiley and Sons. d) The *J*–*V* characteristics of a typical CsPb(I_0.8_Br_0.2_)_3_ PSC before and after LS under AM 1.5G solar illumination (100 mW cm^−2^) in the reverse scan. Adapted with permission.^[^ ^85^ ^]^ Copyright 2021, John Wiley and Sons. *J*–*V* characteristics under different light illumination times for devices based on e) thermally evaporated undoped Spiro‐OMeTAD; and f) versatile solvent annealing assisted thermal evaporation. Adapted with permission.^[^ ^93^ ^]^ Copyright 2022, John Wiley and Sons. *J*–*V* characteristics under illumination for the devices using g) [60]PCBM and h) PTEG‐1 as ETLs. Adapted with permission.^[^ ^102^ ^]^ Copyright 2016, The Royal Society of Chemistry. Factors contributed to the light soaking in PSCs, i) different wavelength dependent *V*_oc_ soaking in PSCs. Adapted with permission.^[^ ^106^ ^]^ Copyright 2017, American Chemical Society.

The origination of enhanced PCE after light soaking can be attributed to the considerable boosting of *V*_oc_ and FF. The dark current is reduced by two orders of magnitude compared with cells lacking light soaking (**Figure** **11**a).^[^ ^92^ ^]^ According to the formula:$V_{\text{oc}}=\frac{KT}{q}\text{ln}\left(1+\frac{I_{\text{sc}}}{I_{\text{Dark}}}\right)$, *V*_oc_ is proportional to ln *I*_sc_/*I*_Dark_ (where *k* is the Boltzmann constant, *T* is the thermodynamic temperature, q is the elementary charge, *I*_sc_ is the short circuit current and *I*_Dark_ is the reverse saturation dark current).^[^ ^92^ ^]^ The depressed dark current would consequently lead to higher *V*_oc_. To better quantify the charge transport property, Du et al. showed up to a 70% reduction of sheet resistance (*R*_sh_) of hole transport materials after 60 min LS, which verifies the positive role of LSE in improving charge transport property (Figure 11b).^[^ ^93^ ^]^ In addition to this, they also studied the influence of LSE on charge transport and recombination in PSCs by electrical impendence spectroscopy (EIS), as exhibited in Figure 11c. Clearly, a substantial increase of the recombination resistance (*R*_rec_) from 610.9 to 7912.9 Ω was observed for the devices under light‐soaking treatment for 60 min. Their result suggested that nonradiative charge recombination in devices could be sufficiently suppressed during long LS process, which explains the gradually increased *V*_oc_ in PSCs under illumination.

To illustrate the LS effect on the device performance of PSCs, Lu et al. fabricated the CsPb(I_0.8_Br_0.2_)_3_ solar cells.^[^ ^85^ ^]^ As shown in Figure 11d, before LS, the device exhibits a relatively low efficiency of 12.58% due to the poor *V*_oc_ and FF. After 30 min of LS, the efficiency reached to 17.16% with improved *V*_oc_ and FF. Xiao et al. also observed the same phenomenon.^[^ ^94^, ^95^ ^]^ Lian et al. constructed favorable ion accumulation in PSCs via illumination to improve the performance of the quasi‐2D PSCs. This design dramatically improves the photo‐carrier collection and enables significant device performance improvement from 14.6% to 19.05%.^[^ ^96^ ^]^ Cai et al. found remarkable LSE in CsPbI_3_‐based solar cells as the PCE increased from 10.8% to 18.3% after 180 s soaking under AM 1.5G sunlight.^[^ ^92^ ^]^ Shao et al. examined the effect of light soaking on the performance of the devices with different perovskite film morphologies.^[^ ^79^ ^]^ After 1.5 h light soaking it is noted that the PCE is more than two times higher than its initial value and important to observe that the light soaking phenomenon is reversible. Additionally, Li et al. reported that a strong LSE occurs in normal n–*i*–p configuration PSCs with the composition of FA_*x*_MA_1−*x*_PbI_3_ (FAMA).^[^ ^97^ ^]^ The PCE of FAMA PSCs increased up to 20.5% from its original 7.5% under continuous light illumination. In addition, the PCE of the Cs^+^‐doped device increased from 18.7% to 22.4% under continuous light illumination.

Zhao et al. suggested that the LSE in PSCs is due to the trap filling by the photogenerated carriers.^[^ ^98^ ^]^ In addition, an in situ doping mechanism has been proposed by Deng et al. to explain this unusual LSE.^[^ ^90^ ^]^ Zhao et al. suggested that light soaking can decrease the surface accumulation of photogenerated charge carriers at the electrode interfaces, and the time‐dependent PL studies suggested that continuous light illumination can reduce the density of charged bulk defects within the perovskite layer through charge‐trapping effects. In addition to this, they expressed that light soaking can decrease the density of charged bulk defects through charge trapping. This can enhance the transport of dissociated charge carriers to the respective electrodes, causing an increase of FF. Furthermore, they indicated that light soaking decreases the bulk polarizations within the perovskite film, with a following impact on reducing the charge dissociation and increase the charge recombination, consequently leading to a decrease of *J*_SC_.^[^ ^98^ ^]^

Additionally, in MHP solar cells, the changes in the properties of hole transport layer (HTL) and electron transport layers (ETLs) can also cause LSE. First, Wen et al. found that with or without Spiro as HTL, the impact on LSE is huge.^[^ ^99^ ^]^ Du et al. found the performance variation under LS can be mainly related to the change in conducting properties of Spiro‐OMeTAD as well as interfacial recombination kinetics (Figure 11e,f).^[^ ^96^ ^]^ Their results showed that LSE depends on the doping status of organic HTL in PSCs. A tenfold efficiency enhancement is realized in the devices based on evaporated undoped HTL caused by LSE. This light‐soaking behavior is attributed to the increased HTL conductivity, enhanced built‐in potential at perovskite/HTL interface, reduced interfacial charge accumulation, and suppressed nonradiative recombination in devices. Moreover, the HTL mobility also determines the LSE strength and kinetics, causing significant variation in photostability of devices. Dong et al. discovered that a low degree of delocalization of the dopants induces intense light‐soaking behavior by showing strong and reversible efficiency fluctuation during light‐dark cycles.^[^ ^100^ ^]^

Qiu et al. systematically investigated the LSE in MHP solar cells adopting metal oxides as ETLs.^[^ ^101^ ^]^ They proved that LS effect commonly occurs in metal oxide‐based MHP solar cells. Shao et al. investigated how ETL with different dielectric constants affects the device performance and the light‐soaking phenomenon in MHP solar cells.^[^ ^102^ ^]^ Fuller pyrrolidine with a triethylene glycol monoethyl ether side chain (PTEG‐1) is employed as ETL in MHP solar cells. The commonly used fullerene derivative PCBM is used as a reference. The device using PTEG‐1 as the electron extraction layers shows a negligible LSE, with a PCE of 15.2% before light soaking and a minor increase to 15.7% after light soaking. In contrast, the device using [60]PCBM as ETL shows severe light soaking, with the PCE improving from 3.8% to 11.7% (Figure 11g,h). Li et al. have systematically investigated the influence of substrate morphology on the LSE of poly[(9,9‐bis(30‐(N,N‐dimethylamino)propyl)‐2,7‐fluorene)‐alt‐2,7‐(9,9‐dioctylfluorene)] (PFN) based PSCs and eliminated this effect by using ultra‐thin PEI gate dielectrics between ITO and PFN electron transport layer. AFM and PL measurements verified the limited ITO/organic interface defects play a crucial role in the light soaking issue.^[^ ^103^ ^]^ Hu et al. concluded that the perovskite/ETL interface is responsible for the light‐soaking effect in direct PSCs. They illustrated that the continuous illumination causes the accumulation of holes at the interface and the bend of the interfacial band, therefore creating an additional electrostatic potential to increase the built‐in voltage, and consequently enhancing the photovoltage.^[^ ^104^ ^]^

Besides, researchers have also observed light effects on different device structures, the LSE is observed in PSCs regardless of device structure. For example, the PCE can ramp up to saturation in only 1 min for TiO_2_‐based normal structure PSCs, whereas at least 5 min is needed in inverted structure PSCs.^[^ ^105^ ^]^ Zhang et al. found that inverted PSCs typically require 5 min to reach a stabilized PCE under light illumination. In addition, they also observed the different effects of different wavelengths of light on the LSE of solar cells (Figure 11i).^[^ ^106^ ^]^ They highlight the ion redistribution in the devices as the underlying reason responsible for this anomalous phenomenon. In contrast, Wang et al. concluded that the light soaking instability of these PSCs mainly originates from the charge accumulation at the TiO_2_/perovskite interface and can be eliminated once the interfacial charge can be suppressed by interfacial modifications to improve charge transport at the interface.^[^ ^107^ ^]^

It is also found that light‐induced performance can increase the PSCs stability. Kobayashi et al. developed encapsulated mesoporous‐carbon perovskite solar mini‐modules that retain more than 92% of their initial performance after 3000 h of damp‐heat aging at 85 °C/relative humidity, while maintaining 90% of the initial value for 3260 h, equivalent to 20‐year stability in outdoor use.^[^ ^89^ ^]^

#### 3.3.2 Photoelectric Detector

**Figure 12:**
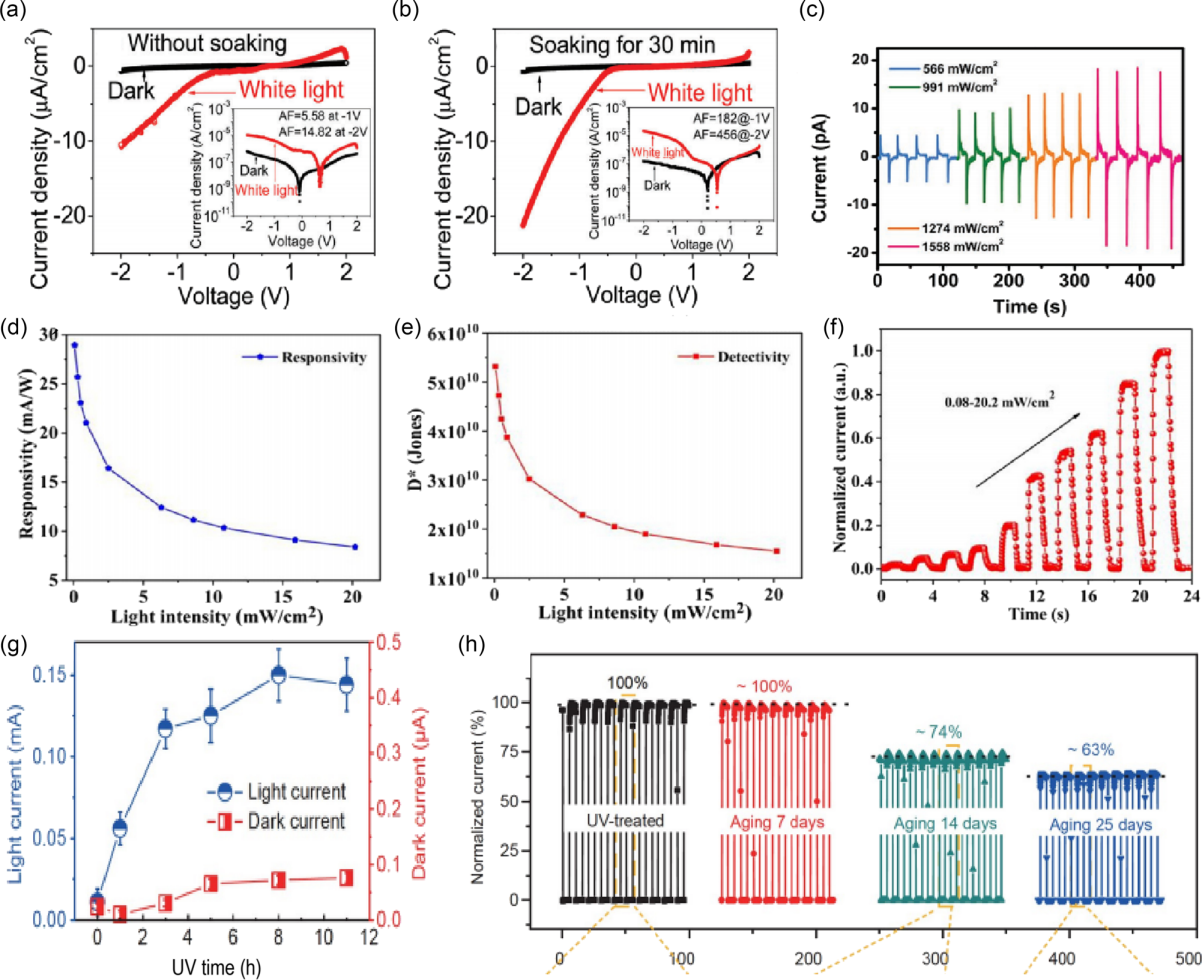
a) current density–voltage (*J*–*V*) characteristics of photodetector device with lateral geometry of Au/MAPbI_3_/C60/BCP/Ag without light soaking under both dark and white light illumination conditions. Adapted with permission.^[^ ^108^ ^]^ Copyright 2018, The Royal Society of Chemistry. b) *J*–*V* characteristics of photodetector device with lateral geometry of Au/MAPbI_3_/C60/BCP/Ag under both dark and white light illumination conditions after light soaking treatment for 30 min. Adapted with permission.^[^ ^108^ ^]^ Copyright 2018, The Royal Society of Chemistry. c) Current–time (*I*–*t*) curves of a photodetector device measured under 1950 nm laser irradiation with different densities at zero bias. Adapted with permission.^[^ ^109^ ^]^ Copyright 2023, John Wiley and Sons. d) Curves of responsivity and e) detectivity of a CsPbClBr_2_ photodetector with different light intensities. Adapted with permission.^[^ ^110^ ^]^ Copyright 2022, American Chemical Society. f) *I*–*t* curve of a CsPbClBr_2_ photodetector under various light intensities (405 nm, 0.08–20.2 mW cm^−2^). Adapted with permission.^[^ ^110^ ^]^ Copyright 2022, American Chemical Society. g) The influence of the UV soaking treatment on the light current and dark current of a Cs_2_AgBiBr_6_ photodetector. Adapted with permission.^[^ ^112^ ^]^ Copyright 2021, Springer Nature. h) The aging stability of the Cs_2_AgBiBr_6_ photodetector after UV soaking treatment. Adapted with permission.^[^ ^112^ ^]^ Copyright 2021, Springer Nature.

The enhancement of photoelectric response by LSE has also been observed in photodetectors. For example, Wu et al. reported giant current amplification in the MAPbI_3_ single crystal photodetectors with a lateral geometry of Au/MAPbI_3_/C_60_/BCP/Ag based on light soaking studies (**Figure** **12**a,b). Upon light soaking for 30 mins, a stabilized device can demonstrate a giant amplification factor reaching 600 at −2 V under weak light illumination (1 mW cm^−2^).^[^ ^108^ ^]^ Tang et al. demonstrated that by introducing the light‐induced pyroelectric effect, the self‐powered and broadband response of a photodetector device can be spanned from ultraviolet (UV) to NIR spectral region (377–1950 nm) (Figure 12c).^[^ ^109^ ^]^

The performance of the photodetector is also related to the intensity of light. The CsPbClBr_2_ film photodetectors based on the band‐to‐band optical transition were fabricated elaborately by Li et al.^[^ ^110^ ^]^ with excellent performance of responsivity of 28.93 mA W^−1^, and a high detectivity of 5.32 × 10^10^ Jones (Figure 12d,e). The current–time (*I*–*t*) curve of the device was tested at a 1 V bias by 405 nm light with various intensities (from 0.08 to 20.2 mW cm^−2^) (Figure 12f), which showed the response current increases with increasing power density. Besides, Wan et al. fabricated 2D RP polycrystalline perovskite (BA)_2_(MA)_3_Pb_4_I_13_ film with excellent crystal orientation by hot‐casting deposition, and a class of 2D hybrid perovskite photodetectors in which the pyrophototronic effect is proposed to achieve temperature and light detection with up to 35 times improved performance.^[^ ^111^ ^]^ The responsivity and detectivity are reported as 12.7 mA W^−1^ and 1.73 × 10^11^ Jones, respectively with an on/off ratio of 3.97 × 10^3^.

The UV stability is one of the critical issues that have hindered further progress of the MHP photodetectors. Yuan et al. found that the UV light‐soaking effect can be significantly positive for improving the performance of a Cs_2_AgBiBr_6_ photodetector and efficiently regulating the response speed (Figure 12g).^[^ ^112^ ^]^. In addition, the photocurrent of the Cs_2_AgBiBr_6_ photodetector exhibits an evident increase with UV soaking treatment, which is significantly boosted from ≈1.0 × 10^−5^ to ≈1.5 × 10^−4^ A. More importantly, the devices exhibited significantly improved operational stability (Figure 12h).

#### 3.3.3 LEDs

**Figure 13:**
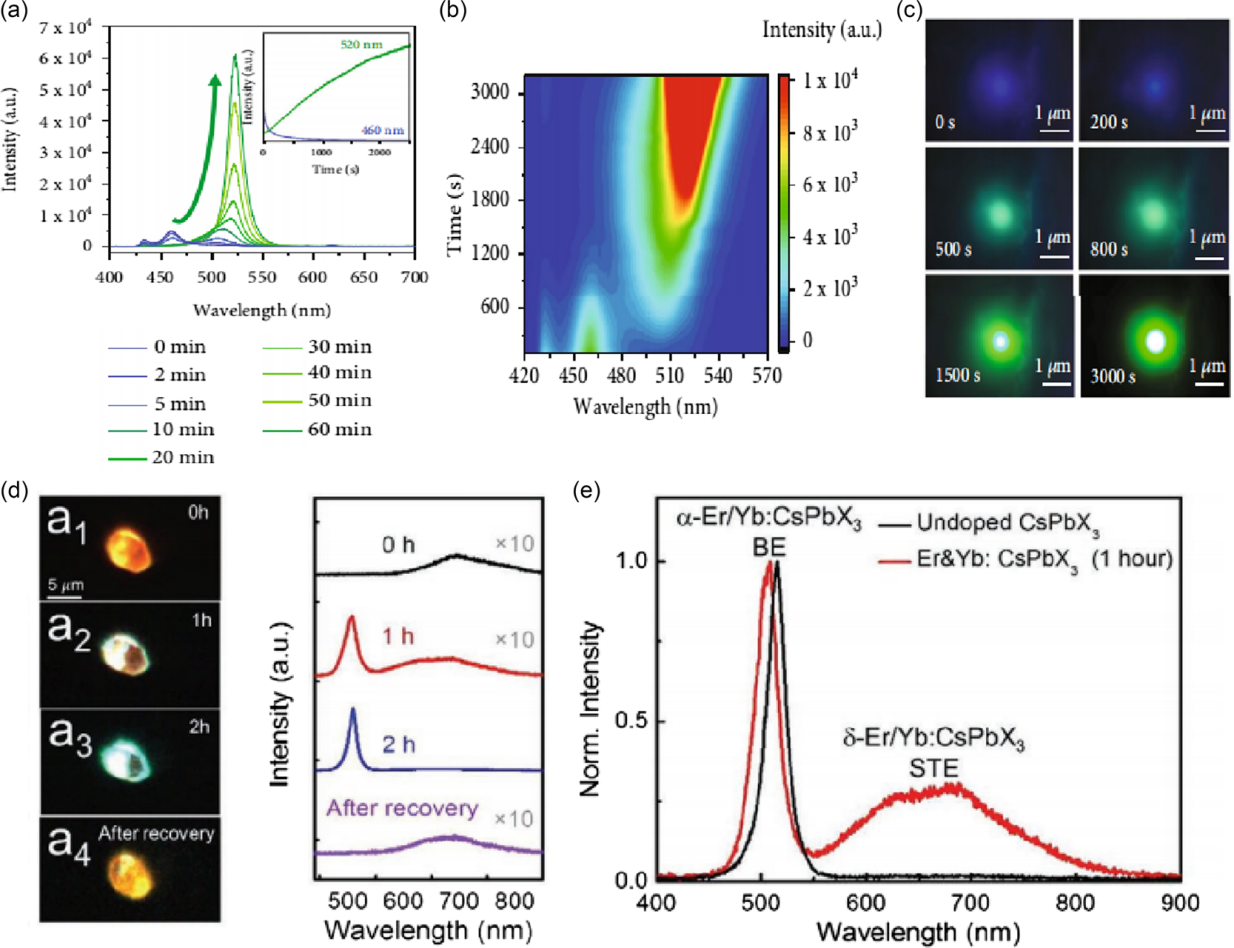
a) PL spectra of the Eu‐MOFs/perovskites composites during UV light soaking. Inset: variations of the PL intensities at 460 nm (blue line) and 520 nm (green line) with irradiation time. Adapted with permission.^[^ ^114^ ^]^ Copyright 2022, The American Association for the Advancement of Science. b) Pseudocolor PL contour mapping during UV light soaking. Adapted with permission.^[^ ^114^ ^]^ Copyright 2022, The American Association for the Advancement of Science. c) Fluorescence microscopic images acquired after different irradiation times. Adapted with permission.^[^ ^114^ ^]^ Copyright 2022, The American Association for the Advancement of Science. d) Optical characterization of typical Er/Yb:CsPb(Cl/Br)_3_ flake. a_1_–a_3_) Real‐color photographs of an individual flake under a laser illumination at 400 nm for 0, 1, and 2 h light illumination. a_4_) After being placed in the dark for 24 h, the emission color returned to orange. Adapted with permission.^[^ ^115^ ^]^ Copyright 2021, John Wiley and Sons. e) PL spectra corresponding to real‐color photographs in panels (a_1_–a_4_) under a constant excitation laser power fluence of 0.35 μJ cm^−2^. Adapted with permission.^[^ ^115^ ^]^ Copyright 2021, John Wiley and Sons.

In recent years, MHP LEDs have emerged as a promising new lighting technology with high external quantum efficiency, color purity, and wavelength tunability, as well as low‐temperature processability. Li et al. systematically discussed the fundamental and engineering aspects of ion‐related issues in LEDs, including the material and origins of ion generation, the mechanisms driving ion migration, characterization approaches for probing ion distributions, the effects of ion migration on device performance and stability, and strategies for ion management.^[^ ^113^ ^]^ The LSE has also been observed in MHP LEDs. For example, Ma et al. synthesized the fluorescent composite containing Eu‐benzene tricarboxylic metal‐organic frameworks (Eu‐BTC MOFs), 0D Cs_4_PbBr_6_, and 3D CsPbBr_3_ by a simple solvent method.^[^ ^114^ ^]^ Interestingly, green (G) fluorescence can be obtained from the Eu‐MOFs/perovskites composites. As shown in **Figures** **13**a,b, exposing the Eu‐MOFs/perovskites composites to a UV laser of 2 mW, the blue PL peak gradually decreases while the green PL peak prominently increases under excitation of 365 nm. Under continuous light soaking, after different irradiation times, the blue fluorescence gradually quenches while the green fluorescence rapidly rises. Finally, the fluorescence color changes from blue to green (Figure 13c).

Yu et al. observed a narrow emission peak at 506 nm with 10 nm linewidth at room temperature and a quite broad peak at 700 nm with 170 nm linewidth in rare earth (Er and Yb) doped all‐inorganic perovskite flakes (CsPb(Cl/Br)_3_).^[^ ^115^ ^]^ The PL bands are ascribed to band‐edge PL of α‐CsPb(Cl/Br)_3_ and self‐trapped excitons of δ‐CsPb (Cl/Br)_3_, respectively. Under light‐soaking, the samples present a color tuning capability spanning from red to green by increasing soaking time, and the emission properties can recover by removing the light‐soaking over a certain recovery time (Figure 13d,e).

## 4 Negative Effects of LSEs

Under light illumination, the reduction of the optical properties of MHPs and the performance of the corresponding devices are usually observed. This sensibility for light originates from the mix ionic‐electronic properties of MHPs. Many reports have shown that ion migration in MHPs is a major factor inducing a series of negative effects. In this chapter, the mechanism of photoinduced ion migration and its negative effects on the film quality, optical properties, and devices of perovskite are systematically described.

### 4.1 Ion Migration and Accumulation

Ion migration plays an important role in composition change such as phase segregation in MHPs and the performance degradation of MHPs‐based devices. Therefore, the inquiry into the inducement of ion migration and the corresponding motional behavior are very critical to the improvement of the stability of MHPs and the related devices. Many studies suggested that the inducement of ion migration is related to the inherent defects of perovskite, weak chemical bonds,^[^ ^116^ ^]^ strongly anharmonic lattice dynamics,^[^ ^117^ ^]^ and reduced activation energy of ions.^[^ ^44^ ^]^ From the viewpoint of mechanical property, MHPs show lower shear modulus which leads to the instability of crystal lattice and consequently feasible ion migration.^[^ ^118^, ^119^ ^]^ Based on a great number of research results, the inducement of ion migration can be considered from the synthesis of perovskite film. The conventional technique to synthesize MHP thin film is based on the solution process methods, in which the formation of a large number of defects is inevitable. Even with the implementation of more sophisticated deposition techniques, defect formation is still unavoidable, as the pathways for ion migration.^[^ ^41^, ^48^ ^]^ Continuous illumination provides the required kinetic energy for ion migration. During light illumination, MHPs are excited by incident light, and the electrons and holes are generated with an elevated carrier temperature, also called hot carriers. These hot carriers equilibrate within hundreds of femtoseconds through carrier–carrier scattering, then relax to the band edge via emitting phonon and transfer their excess electronic energy to the lattice.^[^ ^54^ ^]^ At higher input carrier densities, the carrier temperature increases, through which the thermalization is slow because of a hot‐phonon bottleneck.^[^ ^120^ ^]^ The ions located in the lattice are affected by this electronic energy to escape from lattice and migrate. Barker et al. also suggested that photogenerated hot carriers possess excess energy from illumination thermalizing that provides the required energy for halide ion migration.^[^ ^121^ ^]^ They also exclude the effect of MHPs bulk heating by experiment. Many computational studies have shown that the migration rate is determined by the activation energy. Halide I^−^ ions easily migrate owing to their lower activation energy of 0.08–0.58 eV compared with other constituent ions of MA^+^ with activation energy of 0.46–0.84 eV and Pb^2+^ of 0.8–2.31 eV.^[^ ^21^, ^122^ ^]^

The migration path of ions is directional. Shirzadi et al. reported that there are two directions for halide ion migration including horizontal ion migration and vertical ion migration in alloy perovskites.^[^ ^123^ ^]^ These two types of migration paths are affected by the location of the defects, the horizontal ion migration relies on surface defects to exchange ions between the grains at their interface. The vertical ion migration is more dependent on the point defects within the bulk structure of perovskite. Kim et al. observed that a laser beam induces the horizontal iodine concentration gradient with Gaussian distribution, with the iodine ion migration toward the center of the laser‐illuminated area.^[^ ^124^ ^]^ At the same time, lead ions also migrate from the bulk toward the surface and accumulate near the center of the illuminated area, with their later outward movement to area near the surface.

**Figure 14:**
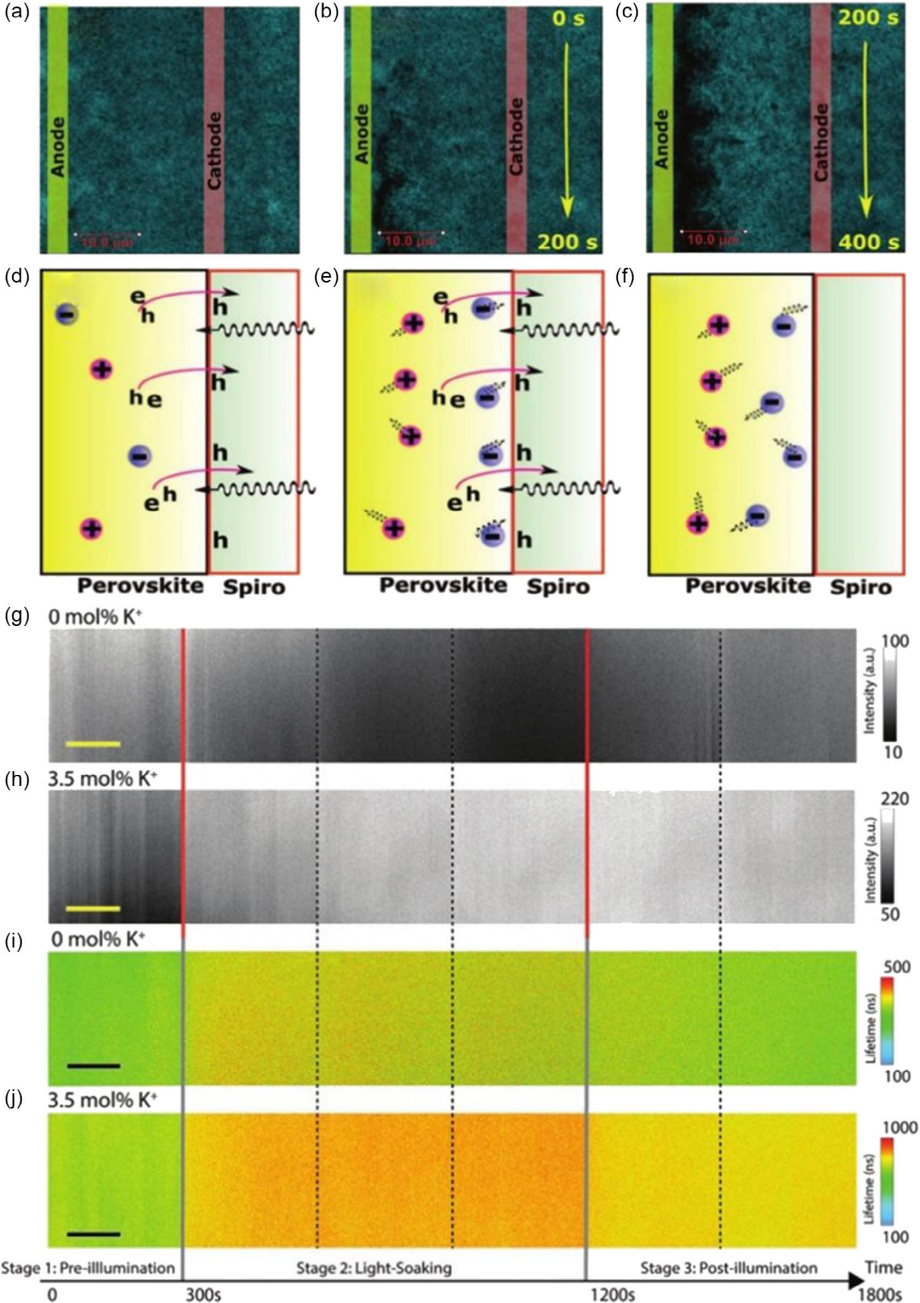
a) Optical image without voltage and b) image with voltage of 10 V (0.5 v μm^−1^) in 200 and c) 400 s. Adapted with permission.^[^ ^127^ ^]^ Copyright 2016, Wiley. Schematic diagrams of ions dynamic processes occurring at perovskite/Spiro interface: d) photogenerated holes transfer into Spiro and an internal electric field establishes in nanosecond timescale, e) negative ions migrate and accumulate at the interface, and f) after switching off the illumination, holes relax quickly and ions drift slowly toward the lower density region. Adapted with permission.^[^ ^127^ ^]^ Copyright 2016, Wiley. g) PL intensity scanning images of 0 mol% K^+^ h) 3.5 mol% K^+^ perovskite films, PL lifetime scanning images of i) 0 mol% K^+^, and j) 3.5 mol% K^+^ perovskite films. Adapted with permission.^[^ ^71^ ^]^ Copyright 2019, John Wiley and Sons.

Ion migration results in ions accumulation near the ion‐blocking interface, which would affect the transport of charge. This is also the main factor leading to the hysteresis in current density–voltage measurement.^[^ ^125^ ^]^ Under illumination, in perovskite‐based photovoltaic devices, photogeneration holes were extracted by hole transport layer such as Spiro‐OMeTAD, leading to a rapidly changing transient electric field. The negative ions were driven to migrate and accumulate to the anode thus affecting carrier extraction and performances of devices. Although ions have a significant influence on charge‐carrier dynamic and photovoltaic properties of MHPs, there is still a lack of effective testing methods to track ion dynamics and distinguish the contribution of ions to photovoltaic properties. Due to its significant impact on carrier dynamic, ion dynamic can be probed by time‐resolved spectroscopic techniques covering a temporal range from picosecond to macroscopic time.^[^ ^126^ ^]^ Wen et al. used specifically designed time‐dependent PL to trace ions dynamic.^[^ ^127^ ^]^ They found the mobile ions during light soaking would accumulate at the interface of CH_3_NH_3_PbI_3_/Spiro‐OMeTAD (**Figure** **14**a–c), impeding the extraction of photogenerated holes from the perovskite layer into Spiro‐OmeTAD (Figure 14d,e). They further stop illumination, the internal electric field disappears, and the ions still remain in the interface due to lower mobility (Figure 14f). In addition, by simultaneously mapping the PL intensity and lifetime of the perovskite film during light soaking, dynamic fluorescence lifetime imaging microscopy also revealed the ions dynamic and interactions with charge‐carrier.^[^ ^71^ ^]^ In Figure 14g,i, the PL intensity and lifetime decrease upon light soaking, indicating the ions migrate and affect carrier transport. This ion migration can be restrained by K^+^ doping, resulting in a monotonic PL enhancement and increase of lifetime (Figure 14h,j). Therefore, these ionic‐carrier interactions determine the photovoltaic properties of MHPs.

### 4.2 Phase Segregation

**Figure 15:**
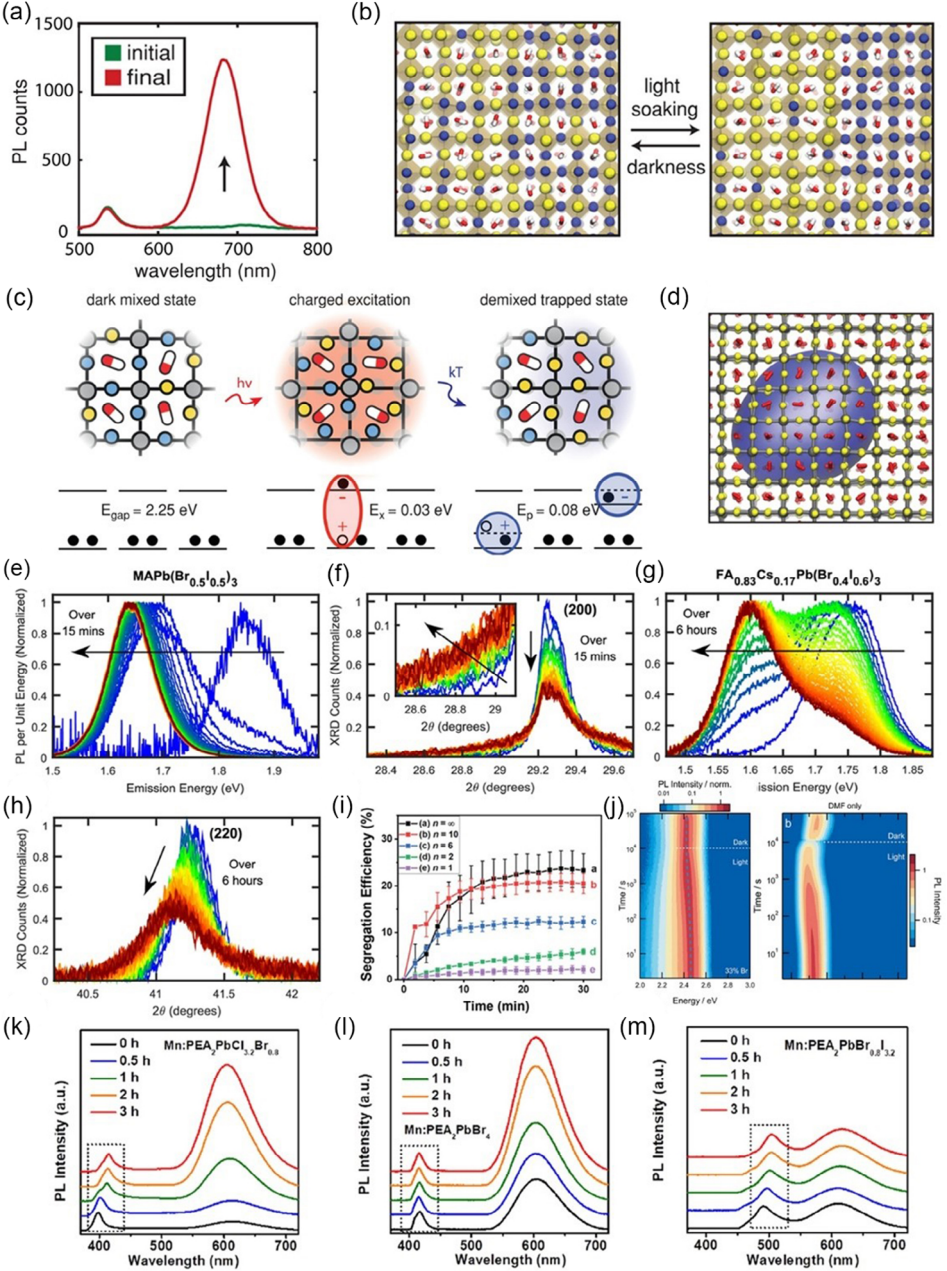
a) PL spectra of MAPb(I_*x*_Br_1−*x*_)_3_ film before (green) and after (red) light soaking for 5 min. Adapted with permission.^[^ ^87^ ^]^ Copyright 2017, American Chemical Society. b) Schematic of phase separation and reversibility in MAPb(I_*x*_Br_1−*x*_)_3_ where yellow and blue spheres represent I^−^ and Br^−^, respectively, the red and white pill shapes represent MA, and the lead atoms are located in the center of the octahedra. Adapted with permission.^[^ ^87^ ^]^ Copyright 2017, American Chemical Society. c) Photoinduced polaron trapping and associated energy scales associated with phase separation. Adapted with permission.^[^ ^87^ ^]^ Copyright 2017, American Chemical Society. d) Snapshot of the 99% isosurface of excess charge density taken from the molecular dynamics simulation. Adapted with permission.^[^ ^87^ ^]^ Copyright 2017, American Chemical Society. e) Normalized PL spectra for an MAPb(Br_0.5_I_0.5_)_3_ thin film, recorded over 15 min of illumination under a light of intensity of 190 mW cm^−1^, wavelength 470 nm. Adapted with permission.^[^ ^128^ ^]^ Copyright 2021, American Chemical Society. f) XRD patterns recorded in situ at the same time and on the MAPb(Br_0.5_I_0.5_)_3_ thin film. The angle axis is scaled to focus on the region around the cubic (200) peak in the recorded XRD data. (Inset) Enlarged region of the low angle tail of the (200) peak.^[^ ^128^ ^]^ Copyright 2021, American Chemical Society. g) Normalized PL spectra for an FA_0.83_Cs_0.17_Pb(Br_0.4_I_0.6_)_3_ thin film recorded over 6 h of illumination under a light of intensity of 190 mW cm^−1^, wavelength 470 nm. Adapted with permission.^[^ ^128^ ^]^ Copyright 2021, American Chemical Society. h) XRD patterns recorded in situ at the same time and on the FA_0.83_Cs_0.17_Pb(Br_0.4_I_0.6_)_3_ thin film. Adapted with permission.^[^ ^128^ ^]^ Copyright 2021, American Chemical Society. i) Efficiency of segregation as a function of photoirradiation time recorded for PEA_2_Pb(Br_0.33_I_0.67_)_4_ films of different dimensionality. Adapted with permission.^[^ ^130^ ^]^ Copyright 2023, American Chemical Society. j) (Left) The normalized PL spectra as a function of time for PEA_2_Pb(Br_0.33_I_0.67_)_4_ film with *n* = 1, and (Right) for *n* = 4. Adapted with permission.^[^ ^131^ ^]^ Copyright 2023, American Chemical Society. The PL spectra of 2D Mn^2+^ doping PEA_2_PbX_4_ microcrystals with k) Cl, Br, l) Br, and m) Br, I halides under 365 nm UV illumination (6 W) with different irradiation times. Adapted with permission.^[^ ^184^ ^]^ Copyright 2023, American Chemical Society.

The phase segregation induced by the illumination broadly has been reported in the mixed‐halide MHPs, with the formation of two different phases of I‐rich and Br‐rich domain with different bandgap.^[^ ^70^ ^]^ **Figure** **15**a shows that upon continued illumination, in the PL spectra of a mixed‐halide MAPb(I_*x*_Br_1−*x*_)_3_ film the 690 nm spectral feature belonging to iodide‐rich region emerges within a certain time scale, indicating the phase segregation.^[^ ^87^ ^]^ Stopping the illumination and storing the MHP thin film in the dark, the iodide‐rich region in the PL spectra of the systems disappears (Figure 15b), which emphasizes the reversibility of the light‐induced phase segregation effect. So far, the mechanisms behind phase segregation have been studied, with a focus on the interaction of several factors of ion migration, polaron formation, and strain release. Ion migration is a mostly proposed factor for phase segregation. Barker et al. reported that the ion migration induces the formation of the iodide‐rich with low‐bandgap regions close to the surface in the MAPb(I_0.1_Br_0.9_)_3_ MHPs film.^[^ ^121^ ^]^ These iodide‐rich regions also lead to the localization of charge carrier.

Illumination can lead to electron–hole pair dissociation and the generation of the free charges. The surrounding lattice of these free charge carriers would deform by electron–phonon coupling. Meanwhile, a polaron can be made through lattice deformation. Lattice deformation is in fact an additional source of strain‐related energy in the system, which needs to be released by phase segregation (Figure 15c,d).^[^ ^87^ ^]^ Shirzadi et al. reported two different phase segregation, inhomogeneous distribution of compositions by horizontal ion migration and ion demixing by vertical ion migration in the Cs_0.08_MA_0.12_FA_0.80_PbI_2.64_Br_0.36_, and Cs_0.3_FA_0.7_PbI_3_ alloy perovskite by statistical analysis of the cathodoluminescence data.^[^ ^123^ ^]^ They suggested that these two processes of ion migration are related to the trapped polarons. Alloyed MHPs in the A‐site show comparable stability compared to single‐cation MHPs. Knight et al. collected dynamic halide segregation in MAPb(Br_0.5_I_0.5_)_3_ (Figure 15e,f) and FA_0.83_Cs_0.17_Pb(Br_0.4_I_0.6_)_3_ (Figure 15g,h) perovskite films by in situ PL spectra.^[^ ^128^ ^]^ Under continuous illumination, the low‐energy PL peak ascribed to iodide‐rich regions is converted from characteristic peak of MAPb(Br_0.5_I_0.5_)_3_, which becomes a fast ionic pathway in MAPb(Br_0.5_I_0.5_)_3_ for facile halide segregation due to the collection of charge carrier into iodide‐rich regions (Figure 15e).^[^ ^128^ ^]^ Whereas in FA_0.83_Cs_0.17_Pb(Br_0.4_I_0.6_)_3_, the PL peak shows a slow overall shift (Figure 15g). From a thermodynamic point of view, iodide‐rich regions with low energy are favorable to be formed in the MAPb(Br_0.5_I_0.5_)_3_.^[^ ^129^ ^]^

It has been proved that low‐dimensional perovskites possess excellent stability in indoor environments compared with conventional 3D perovskites. However, RP 2D perovskite with mixed halide ions also undergoes phase segregation under illumination, similar to 3D ones. Cho et al. found that by reducing the layers of halide perovskites from 3D (*n* = ∞) to quasi‐2D (*n* = 10 − 2), the efficiency and rate of halide ion segregation decrease, with much lower to no phase segregation for the structure with *n* = 1. (Figure 15i)^[^ ^130^ ^]^ By thermodynamic analysis of phase separation, the dimensional dependence of phase separation is related to the activation energy of halide ions. Datta et al. also reported that under illumination, mixed‐halide 2D perovskite PEA_2_Pb(Br_0.33_I_0.67_)_4_ with *n* = 1 only shows a small proportion of iodide‐rich phase compared with *n* = 4 (Figure 15j).^[^ ^131^ ^]^ Storing the system in the dark after illumination, they could not find the phenomenon of redistribution of halide ions. This illumination stability was ascribed to the presence of both iodide‐rich and bromide‐rich octahedra in the mixed‐halide 2D perovskite, with an energy barrier that prevents the movement of the iodide and bromide ions. Although alloying or doping with other ions can enhance the environmental stability of MHPs, partial doping can not necessarily inhibit the phase segregation of 2D perovskites under illumination. In the Mn‐doped 2D PEA_2_PbX_4_ perovskite with varied halogen composition (Cl‐to‐Br and Br‐to‐I), light irradiation also causes halide ion migration (Figure 15k–m).^[^ ^132^ ^]^ Mn^2+^–Mn^2+^ dimers can be formed by annealing above 400 K, under illumination and heating, the Mn‐doped 2D PEA_2_PbX_4_ perovskite can ultimately evolve into PEA_2_PbBr_4_ and PEA_2_MnBr_4_. In addition to the dimensional dependence, the phase segregation also seems to depend on the type of spacer organic cation in 2D perovskites. Mathew et al found that using phenethylammonium (PEA) as the spacer, almost no phase segregation occurs, while the component with butylammonium (BA) RP perovskites can undergo phase segregation.^[^ ^133^ ^]^

### 4.3 Degradation of MHPs

**Figure 16:**
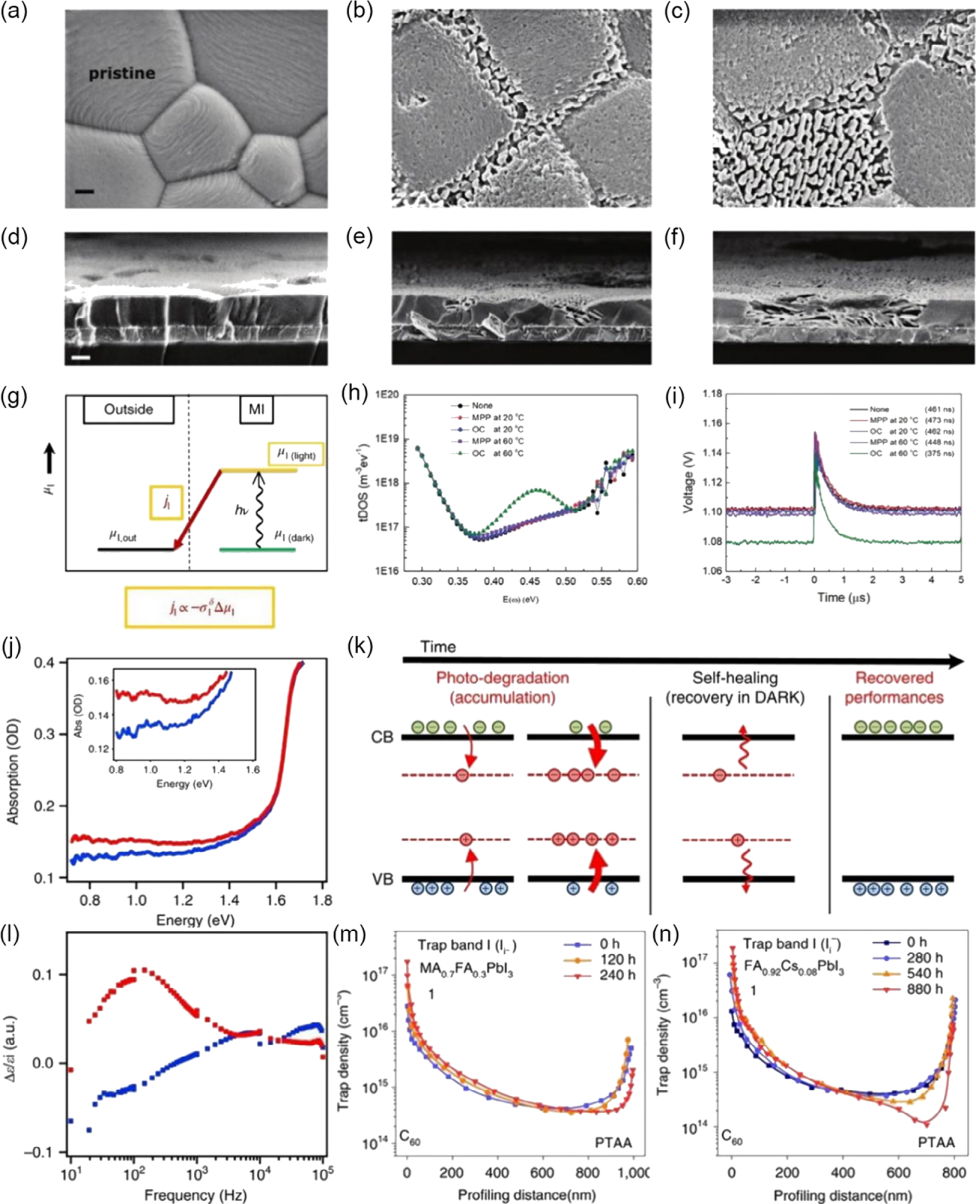
a,d) SEM top view and cross‐section picture of MAPbI_3_ pristine layer, b,e) initial stages of degradation, and c,f) later stages of degradation. The scale bars represent 200 nm. Adapted with permission.^[^ ^135^ ^]^ Copyright 2017, Wiley. g) Schematic of the photodecomposition path. In illustration, the chemical potential of iodine is effectively increased over the equilibrium value in the dark. Adapted with permission.^[^ ^136^ ^]^ Copyright 2017, John Wiley and Sons. The defect formation during degradation of PSCs under illumination. h) Evolution of device trap density of states and i) the transient photovoltage for MAPbI_3_ solar cells after illumination under maximum power point (MPP) at 20 °C, MPP at 60 °C, and open circuit condition at 60 °C for 2 h, respectively. Adapted with permission.^[^ ^137^ ^]^ Copyright 2018, Springer Nature. j) Directly evidence into the deep‐level trap states formed after white light soaking (red line) as compared with steady‐state thin (blue line) by NIR absorption measurement in the MAPbI_3_. The inset is zoomed in region. Adapted with permission.^[^ ^141^ ^]^ Copyright 2021, Springer Nature. k) Schematics of the proposed photocurrent degradation and self‐healing mechanism based on perovskite layer band structure evolution sketching the VB and CB for three situations: during photo‐degradation and accumulation, during recovery in dark and under illumination after self‐healing. The red dotted lines refer to light‐activated meta‐stable trap states that relax in the dark returning the device to its steady state. Arrows sketch how photo‐generated carriers can populate those light‐activated trap states under light or relax in the dark over time.^[^ ^141^ ^]^ Copyright 2021, Springer Nature. l) Low‐frequency real dielectric constant relative charge for a MAPbI_3_ solar cell illumination for 2 h (red) and after recovery in dark (blue), as compared with steady state. Adapted with permission.^[^ ^141^ ^]^ Copyright 2021, Springer Nature. m) Spatial distribution of the trap densities of trap band I in the MA_0.7_FA_0.3_PbI_3_ and n) FA_0.92_Cs_0.08_PbI_3_ solar cell after being illumination at *V*_oc_ condition for different periods of time. Adapted with permission.^[^ ^143^ ^]^ Copyright 2021, The American Association for the Advancement of Science.

The issue of device stability of MHP and their devices has attracted increased attention in recent years. Photodecomposition of MHPs is a common phenomenon about stability, its mechanism is closely related to the external environment, heat, electrical stress, humidity, oxygen, and light, but the mechanism still remains controversial. It has been widely acknowledged that illustration and other external stimulus would lead to a series of chemical reactions. Nickel et al. studied the light‐induced degradation of MAPbI_3_ with and without oxygen. With the presence of oxygen, the CH_3_NH_3_^+^ cation could be deprotonated and resulting in CH_3_NH_3_ and molecular hydrogen.^[^ ^134^ ^]^ Without the presence of oxygen, the dissociation still occurs. But this results from the charge trapping in the N–H antibonding states. Sun et al. observed the degradation of MAPbI_3_ perovskite thin films under light and oxygen.^[^ ^135^ ^]^ As shown in **Figure** **16**a–f, the degradation initiates at grain boundaries, and diffuses into the grain center, with much faster degradation of the smaller grains. The effect of light on ions is also one of the causes of MHPs’ degradation, in fact, the degradation is mostly affected by light rather than oxygen. The specific explanation is reported that under illumination the content of neutral iodine interstitials and effective iodine activity, corresponding ionic conductivity can be enhanced in the MAPbI_3_ perovskite. Figure 16g demonstrates that there is a chemical equilibrium between MAPbI_3_ perovskite and the environment in the dark. Once under illumination, the light‐induced iodine would break the chemical equilibrium. Meanwhile, the chemical potential of iodine can be enhanced, the iodine is extracted into the environment, and ultimately the perovskite decomposes.^[^ ^136^ ^]^

Compared with 3D MHPs, low‐dimensional MHPs have better structural stability. But the degradation still occurs and is connected with larger cations added to the crystal lattice. Zeng et al. found DJ phase 2D perovskite [NH_3_(CH)_3_NH_3_](FA)_*n*−1_Pb_*n*_I_3*n*+1_ can transform to 3D α‐FAPbI_3_ under continuous illumination.^[^ ^137^ ^]^ In this process, the hydrogen bonds between the inorganic layer and the organic diammonium spacers are broken, which causes the dissociation of diammonium spacers. Afterward, α‐FAPbI_3_ is formed by the inorganic layer directly in contact with the adjacent inorganic layer, and also further degrades into PbI_2_ and δ‐FAPbI_3_.

### 4.4 Degradation of MHP‐Based Devices

Stable operation under solar illumination is required for PSCs early commercialization. However, the encapsulated PSCs work merely six months in the realistic operating conditions, due to the unavoidable PSCs degradation under solar illumination.^[^ ^138^ ^]^ Understanding the underlying physical mechanism is crucial to stability improvement. From a single light absorption layer to complex device structure, the effects of composition and structure of PSCs, photothermal, strain, and excess photoexcited charge carrier on photodecomposition should be considered, as well as the electric field and interface between perovskite layer and carrier transport layers or other functional layers in the devices.^[^ ^67^, ^122^, ^139^ ^]^ Chen et al. encapsulated the MAPbI_3_‐based PSCs and investigated the mechanism of light‐induced degradation.^[^ ^140^ ^]^ They found that higher device temperature and excess charges are the dominant sources for degradation of PSCs under illumination because these facilitate the thermodynamic process of defect formation (Figure 16h,i), and the formation of additional nonradiative recombination centers. Deng et al. suggested that the LSE tends to occur at the interface of the perovskite/carrier transporting layer due to the presence of many defects at the interface. Under long light soaking, the performances of MAPbI_3_‐based solar cells decrease caused by ions drifting, and the formation of trap states to undesirably capture the photoexcited carriers.^[^ ^77^ ^]^ Moreover, another degradation mechanism based on changes in the subgap density of states has also been proposed by Nie et al. showing an increasing number of light‐activated meta‐stable states and formation of the charged regions in the bulk of the MAPb(I,Cl)_3_ thin film, accompanied by an increase in the nonradiative recombination, combination of which result in the degradation of the photocurrent. However, when storing PSCs in the dark, the majority of these light‐activated trap states dissipate away (Figure 16j–l).^[^ ^141^ ^]^

The degradation of PSCs also has been proven to be closely related to ion migration and phase segregation.^[^ ^142^ ^]^ The formation of iodide‐rich regions can act as traps to capture the photoexcited charge carriers in the case of phase segregation. Ni et al. reported the starting position of degradation of PSCs by measuring the trap distribution in the MA_0.7_FA_0.3_PbI_3_‐ and FA_0.92_Cs_0.08_PbI_3_‐based solar cells during degradation by the drive‐level capacitance profiling technique.^[^ ^143^ ^]^ Figure 16m,n shows the distribution of I_*i*_^−^ and I_*i*_^+^ in the MA_0.7_FA_0.3_PbI_3_‐ and FA_0.92_Cs_0.08_PbI_3_‐based solar cells under illumination for various durations of light soaking. They found the degradation of the PSCs under illumination starts from the interface between the perovskite and C_60_ layer. The I_*i*_^−^ and I_*i*_^+^ became rich in the interface as a feasible source of trap states for holes and electron charge carriers, respectively.

### 4.5 Current–Voltage (*J*–*V*) Hysteresis

**Figure 17:**
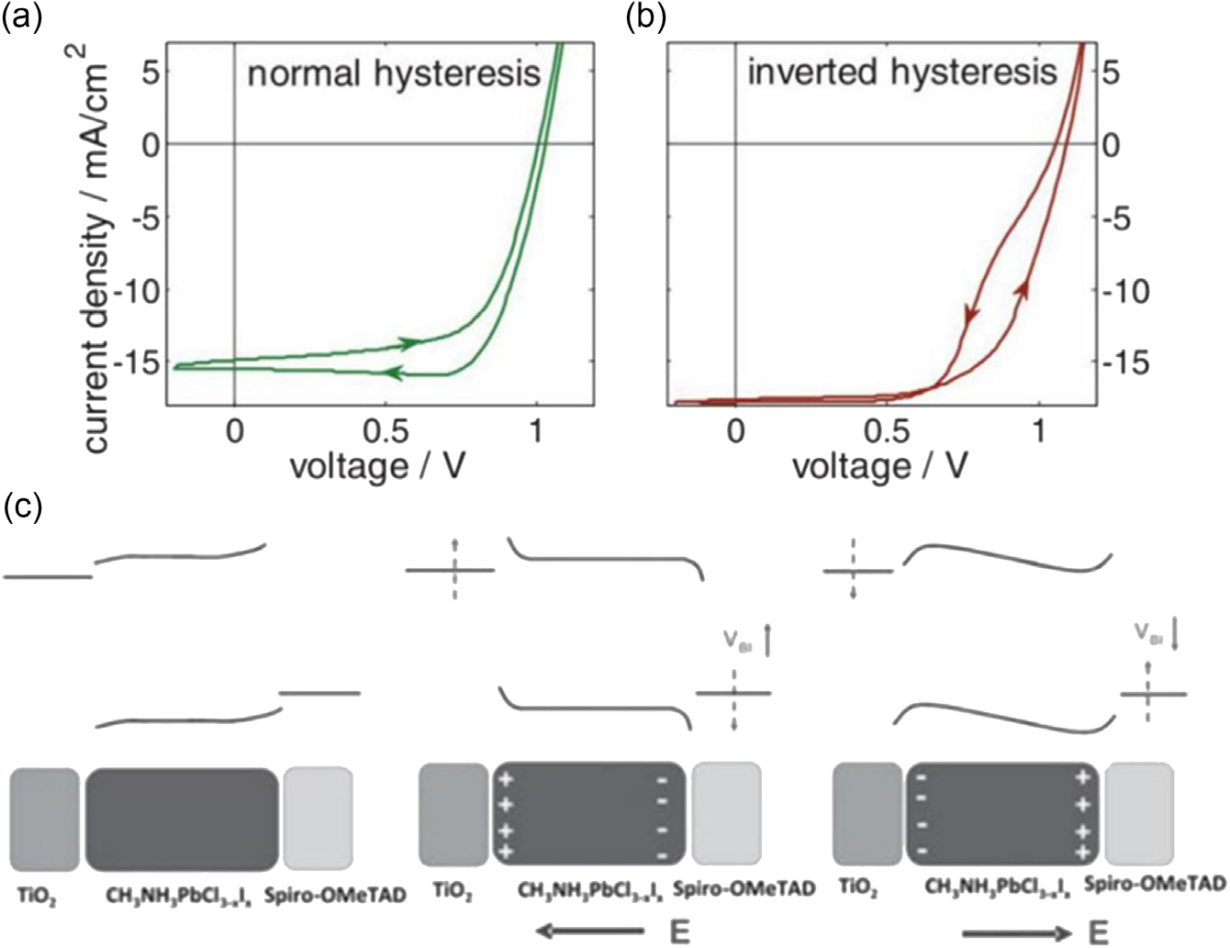
a) Normal and b) inverted hysteresis for a MAPbI_3_ and a mixed PSC, respectively. Adapted with permission.^[^ ^146^ ^]^ Copyright 2016, Wiley. c) Schematic diagrams of band bending in the MAPbCl_3−*x*_I_*x*_ solar cells under electrical fields. The dashed arrows indicate the direction of the effective work function movement. The letter E represents the direction of the external electrical field. Adapted with permission.^[^ ^149^ ^]^ Copyright 2016, Wiley.

PSCs show obviously different current–voltage (*J*–*V*) curves when measured under illumination and voltage sweep. A forward voltage sweeping usually is from negative voltage to positive voltage, the reverse voltage sweeping is performed the other way around (**Figure** **17**a). In most cases, reverse sweep shows a superior performance of PSCs in particular large FF and open circuit compared with forward sweep.^[^ ^144^ ^]^ Interestingly, the “inverted” hysteresis has been observed in the alloy PSCs (Figure 17b) with a decline in the device performance under reverse sweep. Among the reasons explored as the underlying cause for hysteresis phenomenon in PCSs are ion migration,^[^ ^21^, ^145^ ^]^ ionic charge accumulation at the interface,^[^ ^146^ ^]^ deep surface traps,^[^ ^147^ ^]^ ferroelectrics, and giant capacitances.^[^ ^148^ ^]^ The role of ion migration under illumination and electrical fields is widely concerned.^[^ ^146^, ^149^ ^]^ The ions, which usually are iodide ions or interstitials migrate and accumulate to the interfaces of PSCs. In fact, ion migration is often accompanied by the particle movement. The ion migration means the redistribution of ions, which alters the band position and further results in carrier transporting barriers. In contrast, the accumulated ions also change the work function of the constituent layers in PCSs, (Figure 17c) which significantly hampers the photovoltaic performance of the corresponding device.^[^ ^149^ ^]^

### 4.6 PL Blinking

The PL intensity intermittency, also known as PL blinking has been observed in the micrometer‐sized MHPs nanocrystal and polycrystalline films under constant photoexcitation.^[^ ^150^, ^151^ ^]^ Correspondingly, this blinking frequency, which can also be defined as the time interval between the bright (ON), dim (gray), and dark (OFF), has aroused much research interest. Regrettably, it is lossy for PL intensity and quantum yield thus limiting the application of MHPs as LEDs.^[^ ^152^, ^153^, ^154^, ^155^, ^156^ ^]^ These blinking state is affected by the composition of perovskite nanocrystals, the nature of defects, and ion migration in the MHPs.^[^ ^157^ ^]^ Specifically, the origin of PL blinking in the MHPs can be divided into three categories based on different carrier dynamic processes. One is known as nonradiative band‐edge carrier, which is related to the metastable nonradiative recombination centers.^[^ ^158^, ^159^ ^]^ For the case of trapping a charge carrier by a shallow trap state, it relaxes rapidly by the nonradiative process, which results in PL blinking. Another one is that the PL blinking is induced not only by radiative and nonradiative but also by Auger recombination.^[^ ^160^, ^161^, ^162^ ^]^ Thirdly, the capture of hot carriers and their rapid release through nonradiative recombination also generates PL blinking, which is known as hot carrier blinking.^[^ ^163^, ^164^ ^]^

**Figure 18:**
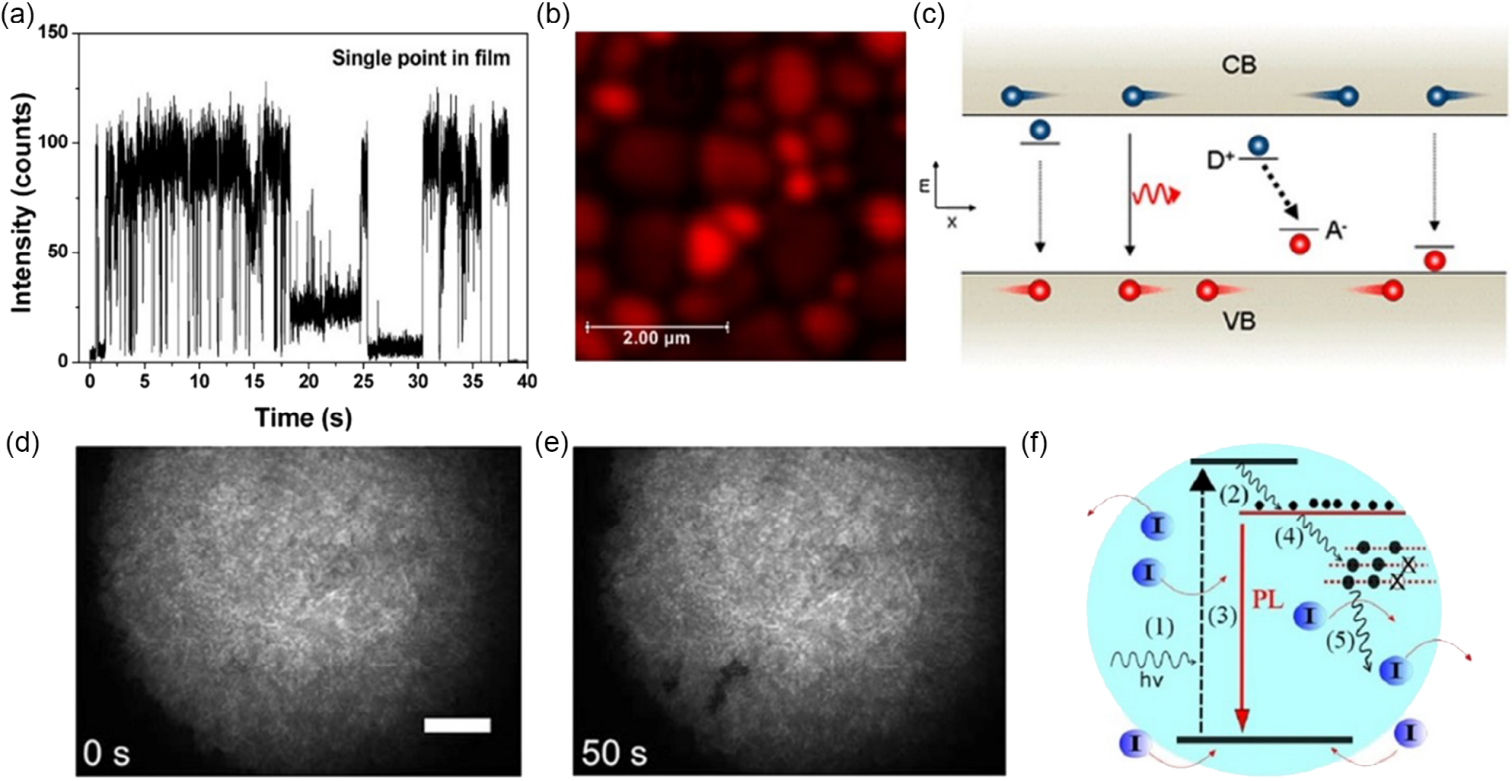
a) Time traces of the fluorescence intensity under a continuous excitation of 470 nm of a single point of the MAPbBr_3_ film. Adapted with permission.^[^ ^14^ ^]^ Copyright 2015, American Chemical Society. b) Fluorescence microscopy image of the MAPbBr_3_ film. Adapted with permission.^[^ ^14^ ^]^ Copyright 2015, American Chemical Society. c) Schematic illustration of super trap as a donor–acceptor pair. The energy diagram schematic showing the sub‐bandgap states formed by the impurities. Adapted with permission.^[^ ^165^ ^]^ Copyright 2017, American Chemical Society. d,e) Temporal evolution of a PL image on the surface of MAPbI_1−*x*_Cl_*x*_ under light illumination of 40 mW cm^−2^ at 532 nm. Adapted with permission.^[^ ^167^ ^]^ Copyright 2017, American Chemical Society. f) Schematic dynamic process of ion activation, migration, and accumulation under higher excitation. Adapted with permission.^[^ ^17^ ^]^ Copyright 2017, John Wiley and Sons.

As **Figure** **18**a,b, Wen et al. observed the PL blinking in the MAPbBr_3_ film, which was dependent on the excitation intensity. Under the excitation density higher than 60 mW cm^−2^, the density and lifetime of the photogenerated mobile charge would increase, and the migration and accumulation at the surface and grain boundaries of the mobile charge also increase further enhancing Auger nonradiative recombination, contributing to the PL blinking. In contrast, PL blinking could not be traced in isolated MAPbBr_3_ nanoparticles due to the absence of mobile charge carriers. So, incremental charge migration plays a key role in PL blinking in the MAPbBr_3_ film.^[^ ^14^ ^]^ Pathoor et al. studied the carrier migration to explain the synchronous multistate PL blinking in the polycrystalline MAPbBr_3_ microcrystals.^[^ ^40^ ^]^ They suggested that the emitted light can be reabsorbed to generate secondary excitons, which promote the capture of the carrier by the metastable nonradiative traps. The primary photogeneration carrier and secondary excitation carrier migration causes the collective quenching of a part of the excited carriers.

In MHPs microcrystal, PL also depends on the size of the microcrystal. Merdasa et al. found that there is no PL in a MAPbI_3_ microcrystal with surface irregularities at a scale of <20 nm.^[^ ^165^ ^]^ The larger size microcrystal with structural homogeneity can emit strong PL under excitation power densities of 1 sun. The MAPbI_3_ crystallites with crystalline domain sizes up to 500 and >100 nm thickness were found to have a strong blinking PL. They suggested the presence of many “supertrap” who is a donor–acceptor pair composed by a hole trap and an electron trap existing in MAPbI_3_ crystal. The supertrap is created by the ionized donor and ionized acceptor complex and efficient nonradiative recombination occurs (thick dashed line). When ionized donor and ionized acceptor are separated, nonradiative recombination is inefficient (thin dashed lines) (Figure 18c). Interestingly, for single CsPbBr_3_ nanocrystal, a memory effect for the PL blinking was found by Hou et al. which links to the movement and reorganization of intrinsic vacancies of single CsPbBr_3_.^[^ ^166^ ^]^

### 4.7 PL Quenching

The PL quenching of MHPs can be seen as the OFF state of PL blinking, as shown in Figure 18d,e, under continuous illumination, where many dark regions appear in the mixed perovskite. This quenching phenomenon is closely related to ion behavior and carrier dynamics. There is a competition between radiative recombination and nonradiative recombination resulting in PL quenching and can be interfered with by photogeneration carriers.^[^ ^167^ ^]^ Usually, the carrier recombination of MHPs is determined by the content of defects and ion species. These defects mostly come from the manufacturing process of perovskite. However, the defect concentration can change due to ion migration or chemical reactions of ions under illumination. Therefore, PL quenching can be associated with the decrease of the photoluminescence quantum yield (PLQY) perovskite devices. In the mixed halide MHPs, the halide ions can migrate to grain boundaries under light illumination and accumulate in these areas, which leads to the formation of interstitial defects with creating deep trap states as nonradiative recombination centers.^[^ ^168^ ^]^ Ion migration not only is induced by lattice thermalization but also by the internal electric field established by the photogeneration holes.^[^ ^127^ ^]^ In addition, due to the decrease of the optical field with penetration depth and illumination range, the diffusion constant of the ion field becomes gradients along the vertical and horizontal directions, which leads to the redistribution of halide ions and the formation of higher defect density regions. The ions can be localized to contribute to nonradiative recombination.^[^ ^167^ ^]^

There are two kinds of behavior for PL quenching, irreversible and reversible. An irreversible PL quenching is mostly induced by trap states and the degradation of perovskite films with the synergistic effects among illumination, moisture, oxygen, and electric field. It depends on the light intensity, where under higher power illumination, the degradation becomes more serious.^[^ ^169^, ^170^ ^]^ The reversibility of PL quenching is more complex, which is affected by the intrinsic relationship between ions behavior, defects, and carrier transport. Hong et al. checked the reversibility of the PL quenching of MAPbI_3_. They found that the PL quenching can reappear with the repeated irradiation, and to recovery the initial state about 30 s, caused by activation/deactivation of traps. The activation process includes a transition between different valence states of interstitial Pb_*i*_ and I_*i*_.^[^ ^171^ ^]^ Chen et al. found the PL quenching in a perovskite film with larger defect density and faster relaxation under higher excitation condition.^[^ ^17^ ^]^ This finding expects that the defects can be activated by higher excitation over energy barrier and as the mobile ions. Similarly, these mobile ions will accumulate at the grain boundary or interfaces, and induce nonradiative recombination of free carriers. And the reversibility of PL quenching may cause by defect cuing and electron relaxation (Figure 17f).^[^ ^17^ ^]^

## 5 Challenges, Opportunities, and Outlooks

The severity of the LSE is reported to vary from laboratory to laboratory and remains the subject of intense debate regarding the underlying mechanism. One of the anomalous phenomena in halide perovskites is that the photogenerated carriers exhibit exceptionally long lifetime and long diffusion length, much longer than those of predicted by the Langevin theory.^[^ ^172^, ^173^ ^]^ Such a long lifetime and diffusion length were previously interpreted as delayed fluorescence or indirect bandgap transition,^[^ ^174^, ^175^, ^176^ ^]^ ferroelectric domains, or photon recycling. The other hypotheses, such as formation of polaron, and screening or giant Rashba effect^[^ ^177^, ^178^ ^]^ have been also proposed to explain the long lifetimes of the charge carriers but their rationality is experimentally disproved.^[^ ^179^, ^180^ ^]^ The aforementioned interpretations exclusively rely on electronic activities while overlooking the ionic contribution of MHPs.^[^ ^181^ ^]^ The escalation of the ion migration under continuous illumination has been widely discussed.^[^ ^46^ ^]^ Nevertheless, these explanations have evidently fallen short in elucidating the phenomena of illumination‐induced fluorescence enhancement and defect tolerance observed in MHPs.^[^ ^15^, ^17^, ^182^, ^183^ ^]^

To date, these hypotheses have not been widely accepted, and they are obviously inconsistent with the experimental phenomena. Therefore, a consistent interpretation has not been yet achieved for the superior optoelectronic properties of halide perovskites, with the lack of establishment of a full picture of the light‐induced carrier dynamic evolution in MHPs. Instead, the observed anomalous effects, such as defect healing^[^ ^15^ ^]^ and defect tolerance,^[^ ^14^ ^]^ are explained simply by phenomenological extensions, rather than physical mechanisms. There are overlapping effects between electrons and ions. Currently, there exists a deficiency in comprehensive physical comprehension, including specific physics principles and computational models to understand the effects of overlaps between electrons and ions. Additionally, there is a scarcity in detection technologies; spectroscopy, while effective in detecting electron transitions, proves inadequate for the study of ions. Therefore, this has greatly restricted the exploration of true potential of perovskite materials and finding the theoretical limit of their resulted optoelectronic devices.

No one seems to have thought about the connection between ions and lattices in perovskite materials and LSE. Anomalous ionic relevant phenomena are observed, while perovskites are confirmed exhibiting mixed ionic‐electronic conduction, such as *I*–*V* hysteresis, phase segregation, PL blinking, PL quenching, degradation/decomposition, illumination‐induced PL enhancement (photobrightening, defect curing/healing), defect tolerance, memory effect, ferromagnetic domain, and so on. Ions in perovskites have multiple roles, such as 1) charge effect: coulomb effect/lattice distortion, and screening effect; 2) defect effect: impact electron‐hole recombination, defect trapping; 3) slow response: larger mass/size, lower mobility, time response typically in seconds; 4) composition effect: phase segregation. So we rationally speculate that the ions in the lattice/sublattice or mobile ions can acquire extra energy during electron−phonon scattering and phonon‐ion coupling.

**Figure 19:**
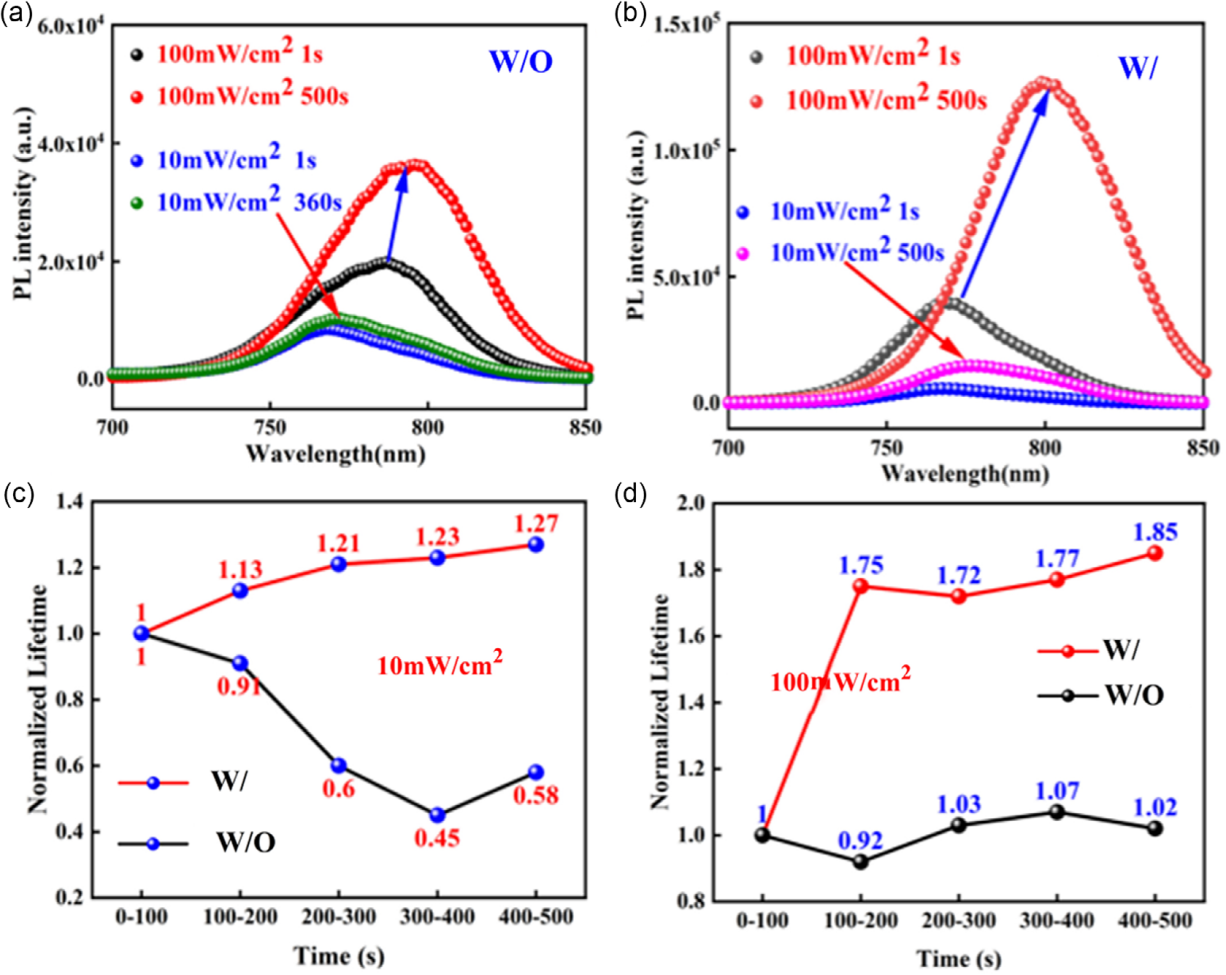
a) The PL intensity increased within 1–500 s under different light intensities of the sample with more surface defects and b) the sample with less surface defects. Normalized data of carrier lifetime of the sample with more surface defects and the sample with less surface defects, under different light intensities, c) 10 mW cm^−2^, and d)100 mW cm^−2^.^[^ ^18^ ^]^ Copyright 2024, American Chemical Society.

While the negative effects have been well studied, the positive effects are poorly understood, mainly due to their high complexity and lack of detection techniques and physical theories. Based on thermodynamics and statistical physics, LSE occurs randomly with statistical probability relative to their threshold that depends on the decomposition, fabrication, ambience, and stimulus conditions. Generally, the positive and negative effects occur simultaneously under given illumination conditions. Localization of hot ions as part of lattice or sublattice results in positive effects, which mostly occur at low illumination. In contrast, at high illumination intensity, the mobile ions are activated, mostly responsible for the negative effect. Moreover, the process is reversible. This phenomenon is reported in our recently published article (as shown in **Figure** **19**). In the same sample, under the same light intensity, the increase of PL intensity and carrier lifetime of the sample (positive effect) and the redshift of the PL peak (caused by phase separation, negative effect) occur simultaneously. Moreover, we have demonstrated that the LSE phenomenon is seriously related to the surface defects of perovskite films and light intensity.^[^ ^18^ ^]^

At low‐intensity illumination, the ions in the lattice obtain limited extra energy but struggle to escape the binding of the lattice as mobile ions. Consequently, the dominant effect at the beginning is the PL enhancement, through which the PL intensity increases, and carrier lifetime prolongs with continuous illumination, followed by a consistent increase of the respective solar cell device performance and stability. These observations have been commonly phenomenally interpreted as dynamic defect tolerance under light illumination. It is reasonable to infer that the positive effects originate from the localized ions (immobile), which result in illumination‐induced PL enhancement and prolonged carrier lifetime, as interpreted as defect tolerance and defect curing/healing. With high‐intensity illumination, ions within the lattice can acquire additional energy, leading to their escape from lattice binding as extra mobile ions and defects. In such scenarios, MHPs predominantly manifest negative effects, including PL quenching and phase segregation, posing a substantial risk of degradation, decomposition, and further phase segregation. Although MHPs have widely reported to have a unique feature of defect tolerance, it is still important to minimize defect density by improving fabrication quality. In the case of low defect density and mobile ions, the illumination‐induced benign effect can significantly overcome defect trapping, leading to high PL efficiency as well as high PCE and stability of solar cells. However, in the case of high defect density under poor fabrication quality, the defect tolerance induced by illumination may not have sufficient capability to overcome strong defect trapping. Simultaneously, the high density of intrinsic mobile ions activated by illumination may pose a high risk of degradation and decomposition.

There is a close relationship between ions and lattices in perovskite structures. The performance characteristics are often influenced by ionic interactions and lattice structures in perovskite materials. It is important to note that the effects of ions and lattice are interrelated in many aspects, where the changes in one aspect may have complex consequences on others. Therefore, understanding the influence of ions and lattice on the performance of MHP systems and the related devices is crucial for further optimizing and designing this class of materials toward their fullest potential. Due to the intricate nature of ionic physics in MHPs and the absence of effective methods for detecting ions, our current understanding of ions in MHPs primarily focuses on mobile ions, while the benign effects of ionic interactions remain poorly understood. Further theoretical and experimental investigations are urgently needed.

## 6 Conclusion

We classify the LSE into positive effects and negative effects to provide a more comprehensive and specific understanding of the LSE. The positive effects include light on the perovskite microstructure and chemical composition, and enhance PL intensity and carrier lifetime. The positive effects of LSE on PSCs, photoelectric detectors, and LEDs are also reviewed. The negative effects include as follows: when MHPs are stimulated by light, ions migration can be induced, which further induces phase separation and degradation of perovskite materials. The performance of corresponding devices would be affected (degradation), with the appearance of *I*–*V* hysteresis. In addition, ion migration also leads to the generation of carrier recombination centers, which results in PL blinking and PL quenching of light‐emitting devices. To sum up, from reviewing the contributed studies, one can say that ions play a significant role in perovskite properties, while more research on ions' behavior, effects, and underlying mechanism is of great importance and highly needed to fully understand the properties of perovskite, prepare high‐quality devices and even expand new application fields. More importantly, we present the current challenges, opportunities, and outlooks, as well as our new perspective. We identify the problems and propose possible solutions to the problems for everyone to discuss.

## Conflict of Interest

The authors declare no conflict of interest.
